# The simultaneous role of porphyrins’ H- and J- aggregates and host–guest chemistry on the fabrication of reversible Dextran-PMMA polymersome

**DOI:** 10.1038/s41598-021-82256-7

**Published:** 2021-02-02

**Authors:** Seyed Milad Safar Sajadi, Sepideh Khoee

**Affiliations:** Polymer Laboratory, School of Chemistry, College of Science, University of Tehran, PO Box 14155 6455, Tehran, Iran

**Keywords:** Supramolecular polymers, Self-assembly, Polymer synthesis

## Abstract

Aggregation-induced quenching of porphyrin molecules as photosensitizer significantly reduces the quantum yield of the singlet oxygen generation, and it is able to decrease the efficacy of photodynamic therapy. We utilized amphiphilic copolymers in this work to precisely control porphyrin H-type and J-type aggregations in water. The amphiphilic copolymer bearing azobenzene, β-cyclodextrin, and porphyrin was successfully synthesized by the atom transfer radical polymerization technique. The azobenzene and β-cyclodextrin complex, as a host–guest supramolecular interaction, has great potential in the design of light-responsive nanocarriers. The amphiphilic block copolymer can be self-assembled into polymersomes, whose application in the generation of singlet oxygen has been also tested. We further demonstrate that, due to the stable H- and J-aggregates of porphyrin, which act as noncovalent cross-linking points, the structure of polymersomes can be reversible under light-stimulus. This formation method has the advantage of allowing for both the encapsulation of hydrophilic and hydrophobic molecules and release upon external light without any distinguishable changes in the structure. Furthermore, the morphology and particle size distribution of the polymersomes were also investigated by using transition electron microscopy, dynamic light scattering, and field emission scanning electron microscopy.

## Introduction

The construction of organic supramolecular assemblies based on porphyrin has attracted great attention in cancer therapy, especially photodynamic therapy (PDT)^1–3^. PDT involves selective accumulation of a photosensitizer and subsequent singlet oxygen production upon photo-irradiation in the presence of oxygen, leading to the cytotoxicity in the tumor cells^4^. Among the promising photosensitizer candidates for PDT, porphyrins have been widely investigated in clinical PDT, e.g. VISUDYNE and PHOTOFRIN, because of their excellent singlet oxygen generation, the quantum yield, and intense absorption^5,6^. However, there are still some limitations in treatments with porphyrin molecules as a photosensitizer. Thus, much effort has been devoted to improve their poor biocompatibility, low hydrophilicity, and high tendency to aggregate via π–π stacking, resulting in quenching of the excitation energy which decreases the quantum yield of the singlet oxygen generation (aggregation-induced quenching)^7,8^. To address these limitations, a variety of strategies have been reported to improve the therapeutic efficacy of porphyrin photosensitizers, such as liposomes and polymeric nanoparticles^9,10^. For example, Zheng et al. reported porphyrin-phospholipid conjugates, self-assembled into porphysomes, a liposome-like structure, providing a high loading capacity and good optical properties with improved performance in photodiagnosis and phototherapy^11^.

The porphyrin photosensitizer incorporated with different biocompatible polymers has shown unique potential, which can improve the photophysical and photochemical properties and further enhance the efficacy of PDT due to its characteristic features. Among different polymers, amphiphilic copolymers have been extensively studied as delivery vehicles for chemotherapeutic drugs utilized for the treatment of cancer, since they can be self-assembled into a rich variety of morphologies, e.g. polymeric nanoparticles, micelles, and polymersomes^12–14^. Among all the nanocarriers, polymersomes have attracted great attention due to their entangled architecture similar to that of lipid vesicles^15,16^. Recent works have focused on designing biocompatible amphiphilic polymers, in which the hydrophilic part is constructed by a natural polymer-like dextran (Dex), modified to possess controlled functional positions on the polymer chain, and the hydrophobic part consists of an amphiphilic polymer, synthesized through controlled radical polymerization processes^17,18^.

The supramolecular strategy consists of π–π stacking, van der Waals forces, and host–guest interactions, making the researchers able to control the self-assembly morphology and the specific molecular packing structure^19^. Among them, Host–guest interactions in an amphiphilic copolymer are versatile for constructing a polymersome. Supramolecular interaction based on host–guest interactions can be formed between the host and guest moieties in a highly-cooperative and controlled manner. Host–guest interactions between azobenzene (Azo) and beta-cyclodextrin (β-CD) have been previously reported as an active connection in a polymersome for responding to the external light stimuli^20,21^. For example, a reversible amphiphilic light-responsive block copolymer was reported to regulate drug release based on Azo and β-CD as guest and host molecules, respectively^22^.

The production of aggregates by π–π interactions as noncovalent cross-linking points has proved to be another smart strategy to improve the function and stability of polymersomes^23,24^. However, the large π-conjugate of porphyrin often undergoes obvious aggregations, decreasing the singlet oxygen generation and seriously limiting the applications of porphyrin in the biomedical field, especially in PDT one. Compared with large π-conjugate, H-type and J-type aggregates with side-by-side and head-to-tail molecular packing, respectively, have attracted more attention for featuring a deeper and stronger absorption^25,26^.

In this study, to decrease the aggregation-induced quenching phenomenon of porphyrin molecules and improve the PDT efficiency, we have synthesized a photo-responsive copolymer via atom transfer radical polymerization (ATRP) of acrylated porphyrin and acrylated β-CD from Azo-containing Dex, as the initiator. The interaction between β-CD rings and Azo, as the host and guest molecules, respectively, was chosen, which was highly desirable for photo-responsive applications due to the reversibility to induce entrapping and releasing molecules upon UV irradiation. Furthermore, by introducing the porphyrin molecules with H & J-aggregations in the polymer structure, robust stability under long-term UV irradiation was given to these polymersome nanocarriers. These porphyrin molecules are able to act as noncovalent cross-linking points, providing a reversible response by the nanocarriers, and also enhance their optical properties for PDT applications.

## Materials and methods

### Materials

Dex (Mw = 15,000–20,000 g mol^−1^), β-CD, 1-naphthaldehyde, 4-hydroxybenzaldehyde, pyrrole, α-bromoisobutyryl bromide (BIBB), 1,1′-carbonyldiimidazole (CDI), sodium cyanoborohydride (NaCNBH_3_), glycidyl methacrylate (GMA), and copper (I) bromide (CuBr) were purchased from Sigma-Aldrich Chemical Company. 3-bromo-1-propanol, di-tert-butyl dicarbonate (Boc_2_O), acryloyl chloride, N,N,N′,N′′,N′′-pentamethyldiethylenetriamine (PMDETA), methyl methacrylate (MMA), triethylamine (Et_3_N), and ethylenediamine were purchased from Merck Chemical Company. Dimethylformamide (DMF), tetrahydrofuran (THF), and dichloromethane (DCM) were dried according to standard procedures before use. All other chemical reagents and solvents were obtained from Merck Chemical Company and used as received unless stated otherwise. 4-phenylazophenol (Azo-OH), N-(2-aminoethyl)-2-bromo-2-methylpropanamide (BIB-NH_2_), 5-(4-hydroxyphenyl)-10,15,20-trinaphthyl porphyrin (Por-OH), and mono-methacrylate modified beta-cyclodextrin (β-CDAc) were prepared as described in the literature^18,27–29^.

### Methods

#### Synthesis of 3-(4-(phenyldiazenyl)phenoxy)propyl 1-imidazolecarboxylate (CI-Azo) as guest part

Azo-OH (1.19 g, 6 mmol), 3-bromo-1-propanol (1.11 g, 8 mmol), and potassium carbonate (1.38 g, 10 mmol) were refluxed in 20 mL of DMF at 80 °C for 24 h. After cooling down to ambient temperature, the mixture was poured into 50 mL of cold distilled water and extracted with chloroform. The organic phase was washed three times with a 10% NaCl aqueous solution and, then, passed over anhydrous MgSO_4_. After filtration, the crude product was obtained by evaporating the solvent. The final yellow powder was purified by using a silica gel column to obtain 4-(3-hydroxypropyloxy)-azobenzene (Azo-C_3_-OH) with a yield of 89% (1.37 g). ^1^H-NMR (500 MHz, CDCl_3_): δ (ppm) 1.78 (s, 1H, CH_2_–O**H**), 2.09 (q, 2H, CH_2_C**H**_2_CH_2_), 3.89 (t, 2H, CH_2_C**H**_2_OH), 4.21 (t, 2H, Ar–O–C**H**_2_CH_2_), 7.02 (d, 2H, *o*-Ar–**H**–OCH_2_), 7.44 (t, 1H, *p*-Ar–**H**–N=N), 7.51 (t, 2H, *m*-Ar–**H**–N=N) 7.90 (q, 4H, *o*-Ar–**H**–N=N–Ar–**H**), FT-IR (neat) ν (cm^−1^): 3266, 3055, 2945, 2867, 1586, 1471, 1385, 1233, 1040, 833.

CDI (0.50 g, 3 mmol) was dissolved in dry DCM (15 mL), and then, the prepared mixture cooled down to 0 °C by using an ice bath under an inert nitrogen atmosphere. Subsequently, a mixture of Azo-C_3_–OH (0.5 g) and dry DCM (10 mL) was added dropwise to the solution over 1 h. The foregoing mixture was stirred at 0 °C for an additional 1 h, followed by 24 h stirring at ambient temperature. The reaction mixture was diluted with DCM (20 mL), followed by being washed with distilled water, dried over anhydrous MgSO_4_, and concentrated under vacuum to afford 0.635 g of a yellow solid with 93% yield. ^1^H-NMR (500 MHz, CDCl_3_): δ (ppm) 2.36 (q, 2H, CH_2_C**H**_2_CH_2_), 4.22 (t, 2H, Ar-OC**H**_2_CH_3_), 4.72 (t, 2H, CH_2_C**H**_2_–OC=O), 6.99 (d, 2H, *o*-Ar–**H**–OCH_2_), 7.20 (d, 1H, CH=N–C**H**=CH), 7.44 (t, 1H, *p*-Ar–**H**–N=N), 7.50 (m, 3H, *m*-Ar–**H**–N=N and (C=O)–N–C**H**=CH), 7.90 (q, 4H, *o*-Ar–**H**–N=N–Ar–**H**), 8.50 (s, 1H, N–C**H**–N), FT-IR (neat) ν (cm^−1^): 2970, 1747, 1594, 1476, 1407, 1245, 759.

#### Synthesis of azobenzene grafted bromoisobutyramide-ended dextran (Azo-grafted Dex-Br) as a macroinitiator

The synthesis of bromoisobutyramide-ended dextran (Dex-Br) was performed according to the literature with minor modifications^18^. Accordingly, in a three-necked round-bottom flask, Dex (2.6 g, 0.15 mmol), sodium cyanoborohydride (0.75 g, 12 mmol), Et_3_N (0.56 mL, 4 mmol), and BIB-NH_2_·HCl (1.0 g, 4 mmol) were dissolved in DMSO (10 mL). Then, the mixture was strongly stirred at 65 °C for 3 days under an inert atmosphere of nitrogen gas. Next, the mixture of the reaction was purified through dialysis against distilled water in a dialysis bag (Sigma-Aldrich) with a molecular weight cut-off (MWCO) of 12 kDa and lyophilized for 3 days. After lyophilization, Dex-Br was obtained as a white powder with a yield of 85% (2.25 g).

Dex-Br (1 g), CI-Azo (0.2 g), and Et_3_N (three drops) were mixed in anhydrous DMSO (8 mL) in a dry round-bottom flask, and subsequently, the mixture was stirred for 2 days at 60 °C under an inert atmosphere. The reaction mixture was precipitated in acetone (50 mL), and then, the solid product was filtered, washed with acetone, and dried to obtain Azo-grafted Dex-Br as a yellow solid with a yield of 72% (0.84 g).

#### Synthesis of porphyrin acrylate (PorAc)

The procedure was similar to the synthesis of Azo-C_3_–OH, except that the Azo-OH was replaced in this route by Por-OH with the yield of 89% (1.37 g). ^1^H-NMR (500 MHz, CDCl_3_): δ (ppm) − 2.34 (s, 2H, N**H** pyrrole), 2.23 (q, 2H, CH_2_C**H**_2_CH_2_), 4.03 (t, 2H, CH_2_C**H**_2_O–C=O), 4.4 (t, 2H, Ar–O–C**H**_2_CH_2_), 7.07–7.25 (m, 8H), 7.43–7.51 (m, 3H), 7.79–7.88 (m, 3H), 8.06–8.16 (m, 5H), 8.23–8.32 (m, 6H), 8.43–8.51 (m, 4H), 8.55–8.61 (m, 2H), 8.76–8.82 (m, 2H).

Acryloyl chloride (0.1 mL, 1.2 mmol) was dissolved in dry THF (5 mL) and added dropwise to the stirring mixture of Et_3_N (0.17 mL, 1.2 mmol) and Por-C_3_–OH (0.1 g, 0.12 mmol) in dry THF (10 mL) over 1 h at 0 °C. The reaction mixture was stirred overnight at 25 °C, and then, the solvent was evaporated under reduced pressure. In the next step, distilled water (20 mL) was added into the residue and the product was extracted three times with DCM (20 mL). After removing DCM, the crude product was purified by using a silica gel column with DCM, as the eluent, to obtain 90 mg of PorAc monomer with 85% yield. ^1^H-NMR (500 MHz, CDCl_3_): δ (ppm) − 2.30 (s, 2H, N**H** pyrrole), 2.37 (q, 2H, CH_2_C**H**_2_CH_2_), 4.36 (t, 2H, CH_2_C**H**_2_O–C=O), 4.54 (t, 2H, Ar–O–C**H**_2_CH_2_), 5.90 (d, 1H, (C=O)–CH=C**H**_2_), 6.23 (dd, 1H, (C=O)–C**H**=CH_2_), 6.51 (d, 1H, (C=O)–CH=C**H**_2_), 7.09–7.28 (m, 8H), 7.46–7.53 (m, 3H), 7.80–7.90 (m, 3H), 8.10–8.17 (m, 5H), 8.24–8.35 (m, 6H), 8.46–8.54 (m, 4H), 8.58–8.64 (m, 2H), 8.79–8.85 (m, 2H).

#### Synthesis of b-(Azo-grafted Dex)-b((MMA)-r-(β-CDAc)-r-(PorAc))

In a Schlenk tube, Azo-grafted Dex-Br macroinitiator (20 mg), β-CDAc (12 mg), PorAc (9 mg), and MMA (14 mg), as a comonomer, were dissolved in degassed DMF (6 mL). In another Schlenk tube, CuBr (1 mg) and PMDETA (1 µL) were also dissolved in 4 mL of degassed DMF. After conducting five freeze–pump–thaw cycles through the Schlenk line, the second solution was added into the first degassed solution tube through the cannula under an inert nitrogen atmosphere. The reaction mixture was allowed to be stirred for 6 h at 75 °C. After cooling the mixture, purification was applied via dialysis against distilled water by using a dialysis bag with MWCO of 12 kDa and lyophilized for 3 days. After lyophilization, the amphiphilic diblock copolymer was obtained as a pink powder.

#### Self-assembly of b-(Azo-grafted Dex)-b((MMA)-r-(β-CDAc)-r-(PorAc))

Nanosized polymersomes were obtained by the following steps. Firstly, b-(Azo-grafted Dex)-b((MMA)-r-(β-CDAc)-r-(PorAc)) (0.2 mg) was dissolved in THF (1 mL) by using a magnetic stirrer (1000 rpm) in a test tube at 25 °C. In order to control the water flow rate during the injection, deionized water was added dropwise into the polymer solution up to a total volume of 4 mL by using a syringe pump. Finally, the organic solvent was partially removed via evaporation under stirring for 2 days at 25 °C. The resulting sample was solidified by a fast liquid nitrogen cold trap, followed by the freeze-drying process.

#### Singlet oxygen detection

To assess the ability of the prepared polymersomes in the generation of singlet oxygen, a chemical method was performed by using indocyanine green (ICG), as a reactive oxygen species indicator, and monitored by UV–vis spectroscopy. A solution containing polymersome (0.1 mg/mL) in DMF was mixed with 20 μL of ICG water solution (1 mg/mL), and subsequently, the mixture was irradiated with a 430 nm light source for different time intervals. The absorption intensity of ICG was monitored every three min intervals. The same procedure was also done with the ICG solution in DMF as a control test.

#### Preparation of drug-loaded nanoparticles

For the preparation of drug-loaded nanoparticles, a solution containing 1 mg of polymersome in DMSO (1 mL) was separately mixed with 0.1 mg of quercetin (Q), Fluorouracil (5-FU), and a combination of Q and 5-FU. Each of the above-mentioned mixtures of the polymersome and drugs was stirred gently for 48 h at room temperature to allow drugs to be absorbed into the nanoparticles. Drug-loaded polymersomes were obtained after lyophilization and washed once with ethanol to remove the unloaded drugs, followed by being freeze-dried for further analyses. Drug loading and encapsulation efficiencies of the prepared polymersomes were measured by UV–Vis spectroscopy. To measure the amount of encapsulated drugs, the amount of non-encapsulated drugs was initially measured and, then, subtracted from the total amount of the feeding drugs. The absorbance of the drugs was determined at 380 nm for Q and 268 nm for 5-FU. The absorbance values were converted into the quantity of drugs encapsulated within nanoparticles by using the standard calibration curve. Next, the drug loading (DL) and encapsulation efficiency (EE) were calculated by using the following equation:

1 $$ DL\left( \% \right) = \frac{{Weight\;of\;feeding\;drug - weight\;of\;non{ - }encapsulated\;drug}}{Weight\;of\;nanoparticles} \times 100 $$

2 $$ EE\left( \% \right) = \frac{{Weight\;of\;feeding\;drug - weight\;of\;non{ - }encapsulated\;drug}}{Weight\;of\;feeding\;drug} \times 100 $$

#### In-vitro release of drugs from nanocarriers

Equal amounts of the drug-loaded polymersomes (1 mg) were dispersed separately in 3 mL phosphate buffer solutions (PBS, pH = 7.4). Subsequently, all the samples were transferred into dialysis bags with a molecular weight cut-off (MWCO) of 12,000 Da, which were sealed at both ends and, then, immersed in a 10 mL PBS buffer solution. All drug-loaded samples were incubated at 37 °C and at determined time intervals. Next, 2 mL of the release medium was withdrawn by using a syringe and replaced with the same volume of fresh PBS. A similar procedure was also repeated in the presence of a UV light source. The absorbance of the collected samples was measured by UV–Vis spectroscopy at wavelengths of 203 nm and 265 nm for Q and 5-FU, respectively. The percentage of drug release was calculated according to Eq. (3), where M_0_ and M_t_ are related to the amount of the loaded and released drugs, respectively.

3 $$ Drug\;release\;\left( \% \right) = M_{t} {/}M_{0} \times 100 $$

### Characterization

Proton nuclear magnetic resonance (^1^H-NMR) spectroscopy and 2D NOESY NMR experiment were performed on a 500 MHz Varian Innova spectrometer to determine the chemical structure of the synthesized molecules and polymers. The spectra were processed by using the TopSpin 3.5pl6 and MestReNova v12 software. Gel permeation chromatography (GPC) measurements of the synthesized polymers were conducted on an Agilent 1260 infinity system operating in THF, as the eluent, which was equipped with a refractive index detector (RID) and variable wavelength detector (VWD) and calibrated by linear polystyrene samples. The mean hydrodynamic diameters (D_h_) were determined by using a Horiba SZ-100 Nanoparticle Size instrument equipped with a diode-pumped laser at 532 nm. DLS measurements were conducted on the freshly prepared nanoparticles dispersion (1 mL). All the DLS measurements were performed at 25 °C and repeated three times. UV–visible spectra were recorded on a Jasco V-710 spectrometer equipped with a temperature control cell at 25 °C in DMSO in the range of 200–700 nm by using quartz cuvettes. Field Emission Scanning Electron Microscopy (FESEM) images were obtained by using a MIRA3TESCAN-XMU field-emission scanning electron microscope operated at 15 kV, and Fluorescence Microscopy images were generated by using a Zeiss Axioplan 2 microscope controlled by MetaMorph image acquisition system. A Zeiss LEO 906 TEM instrument was employed to perform the Transmission Electron Microscopy (TEM) analysis and study the selected area electron diffraction (SAED) pattern.

## Results and discussion

**Figure 1:**
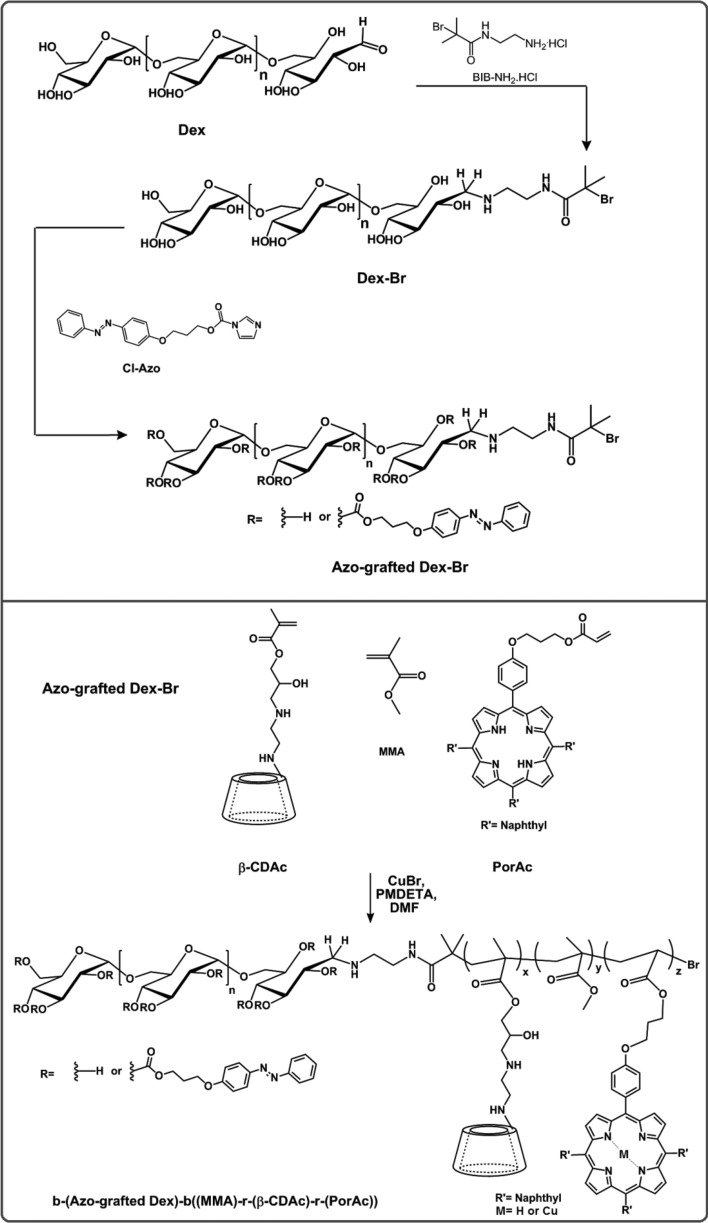
Schematic representation for the synthesis of b-(Azo-grafted Dex)-b((MMA)-r-(β-CDAc)-r-(PorAc)).

The design and synthesis of a reversible host–guest nanocarrier can provide a method for on-demand release of a therapeutic cargo, without degrading the nanocarrier structure. In this study, we synthesized an amphiphilic copolymer based on dextran/poly(methyl methacrylate) that contains Azo and *β-*CD groups for supramolecular interactions, as well as porphyrin units for dedicating the stability to these carriers as a consequence of π–π stacking (Fig. 1). Detailed synthesis description and characterization of each structure were described in the following.

### Synthesis of the hydrophilic block containing Azo groups

#### Synthesis of CI-Azo

CI-Azo was prepared in three steps (Fig. S1), as follows: the first step was the synthesis of Azo-OH by using aniline and phenol as starting materials through the diazonium salt route (generated in situ); in the next step, Azo-C_3_–OH was synthesized according to the nucleophilic substitution reaction by using Azo-OH and 3-bromo-1-propanol as raw materials in the presence of K_2_CO_3_; and the final step involved the activation of free hydroxyl groups as a reactive carbamate linkage by using an excess amount of CDI. The structure of the prepared materials is confirmed by ^1^H-NMR (Figures S2–S4). In the ^1^H-NMR spectrum of Azo-OH, the signals appeared at δ = 6.95–7.9 ppm are related to the protons on the aromatic rings, and the other up-field singlet at δ = 5.35 ppm is assigned to the proton of the hydroxyl group of phenol (Fig. S2). By the reaction of the hydroxyl group on Azo-OH with 3-bromo-1-propanol, the aromatic protons of Azo-C_3_–OH remained relatively unchanged. However, the appearance of aliphatic protons at 2.10 (f), 3.91 (g), and 4.22 (e) ppm, related to the propanol residue, and disappearance of aromatic hydroxyl groups revealed that the propanol moiety was coupled to the Azo compound (Fig. S3). Azo-C_3_–OH was reacted with CDI, to convert the hydroxyl end group to a good leaving group. Furthermore, the ^1^H-NMR spectrum of CI-Azo shows the entire Azo-C_3_–OH characteristic peaks as well as the appearance of CDI protons resonances at 7.18 (k), 7.5 (i), and 8.50 (h) ppm (Fig. S4).

#### Synthesis of Azo-grafted Dex-Br (ATRP macroinitiator)

First of all, the ATRP initiator (BIB-NH_2_) with two different functional groups at two ends, i.e. amine and bromine, was prepared in three steps (Fig. S5), and the product of each step was characterized by using ^1^H-NMR (Figures S6–S8). Boc-NH_2_, which was prepared from the reaction between ethylenediamine and Boc_2_O, exhibits the characteristic peak of tert-butyl group of the Boc residue at 1.41 (e) ppm (Fig. S6). Amidation of one of the two amine groups has also resulted in a peak at 2 ppm and a downfield peak at 5.17 ppm related to amine and amide protons, respectively. Fig. S7 illustrates the ^1^H-NMR spectrum of BIB-N–N-Boc obtained from the reaction of Boc-NH_2_ with BIBB. The peak related to 2-bromoisobutyryl methyl protons at 1.98 ppm appeared at a different chemical shift from the BOC methyl protons (1.41 ppm), which revealed that the reaction was successfully performed. After Boc deprotection under acidic conditions, the Boc methyl protons of BIB-NH_2_.HCl disappeared completely (Fig. S8). Then, the primary amine of BIB-NH_2_ reacted with the terminal anomeric aldehyde of dextran in the presence of sodium cyanoborohydride, as the reducing agent of the imine group, to produce Dex-Br (Fig. S9a). CI-Azo was then conjugated to Dex-Br via a carbonate bond between hydroxyl groups of the Dex-Br and carbonyl groups of CI-Azo to obtain Azo-modified dextran (Azo-grafted Dex-Br), as shown in Fig. S9b. The successful attachment of the Azo functional groups was confirmed by ^1^H-NMR spectroscopy. The ^1^H-NMR spectrum of the commercial Dex, Dex-Br, and Azo-grafted Dex-Br with all assignments are shown in Fig. S10. According to ^1^H-NMR, Azo functional groups have been successfully conjugated to the Dex chain, by an average of about 9 groups per chain. Pendant Azo groups play the guest role which has supramolecular interaction with β-CD, as the host molecule, to control the structure and drug release of the final polymersome.

### Synthesis of the hydrophobic block containing porphyrin and β-cyclodextrin pendant groups

#### Synthesis of PorAc monomer

PorAc monomer was prepared by using three sequence steps (Fig. S11). In the first step, Por-OH was synthesized through condensation of commercially available pyrrole, 1-naphthaldehyde, and 4-hydroxybenzaldehyde as raw materials. Then, Por-C_3_–OH was prepared from the reaction of Por-OH and 3-bromo-1-propanol in the presence of potassium carbonate. Finally, the PorAc monomer was synthesized via an esterification reaction between Por-C_3_–OH and acryloyl chloride. Their structures are confirmed by ^1^H-NMR (Figures S12–S14). In the ^1^H-NMR spectrum of Por-OH, the signals at δ = 7.05–8.83 ppm are assigned to aromatic and pyrrole ring protons, and the other signal at δ = −2.34 ppm is related to the internal pyrrole-NH proton (Fig. S12). After the reaction of phenol hydroxyl on Por-OH with 3-bromo-1-propanol, the aromatic protons of Por-C_3_–OH remained relatively unchanged, and new peaks appeared at δ = 2.23 (n), 4.03 (o) and, 4.40 (p) ppm, which belonged to methylene protons, as shown in Fig. S13. PorAc was prepared by the coupling reaction between Por-C_3_–OH and acryloyl chloride, and the ^1^H-NMR spectrum of PorAc was shown in Fig. S14. Besides the signals at δ = 7.09–8.85 ppm and -2.30 ppm, assigned to aromatic and pyrrole ring protons, and the signals at 2.37 ppm, 4.36 ppm, and 4.54 ppm, assigned to the methylene protons, the signals appeared at 5.90 ppm (q), 6.23 ppm (p), 6.51 ppm (q) are attributed to the protons of the acrylate group.

#### Synthesis of b-(Azo-grafted Dex)-b((MMA)-r-(β-CDAc)-r-(PorAc))

β-CD acrylate (β-CDAc), as the comonomer of the hydrophobic block, was synthesized in three steps (Fig. S15) and characterized by ^1^H-NMR spectroscopy (Figures S16–S18). In the ^1^H-NMR spectrum of tosyl-β-CD (Fig. S16), the peaks at δ = 7.74 (c), 7.42 (b), and 2.42 (a) ppm are attributed to the protons of the attached tosyl group, suggesting the successful synthesis of tosyl-β-CD. After the reaction of tosyl-β-CD with ethylenediamine, it is clear in the spectrum of the EDA-β-CD product that some new peaks have appeared at the 2.60–2.80 (a and b) ppm region, which are related to the protons of methylene groups (Fig. S17). Carrying out of this reaction led to the complete disappearance of the tosyl group corresponding peaks. EDA-β-CD was reacted with GMA to introduce the methacrylate group to β-CD via the ring-opening reaction of the epoxy group with the primary amine of EDA-β-CD. The ^1^H-NMR spectrum of β-CDAc shows the entire characteristic peaks of EDA-β-CD as well as GMA protons resonances at δ = 1.93 (f), 5.74 (g), and 6.10 (g) ppm (Fig. S18).

The hydrophobic block containing acrylated β-CD, acrylated porphyrin, and MMA was polymerized from the terminal Br group of Dex-Br macroinitiator. The reaction was carried out in DMF by using the CuBr/PMDETA catalyst complex. Final amphiphilic copolymers [b-(Azo-grafted Dex)-b((MMA)-r-(β-CDAc)-r-(PorAc))] were prepared, as exhibited in Fig. S19. The successful attachment of β-CD, Por, and MMA to the macroinitiator was confirmed by ^1^H-NMR spectroscopy. The ^1^H-NMR spectrum of the b-(Azo-grafted Dex)-b((MMA)-r-(β-CDAc)-r-(PorAc)) block copolymer is exhibited in Fig. S20. The characteristic signals at δ = 6.75–8.56 (Ar and Ar′) are ascribed to the aromatic protons of the azobenzene and porphyrin moiety, and the signals ranging from 5.63 to 5.88 ppm are assigned to OH-2,3 of β-CD^30,31^. The peaks corresponding to methyl and methylene protons (k) of the hydrophobic block appeared in the δ = 0.59–2.25 ppm region, demonstrating that the b-(Azo-grafted Dex)-b((MMA)-r-(β-CDAc)-r-(PorAc)) block copolymer was successfully synthesized. Furthermore, the other peaks are also exhibited in Fig. S20. The weird problem was that after carrying out ATRP polymerization, one of the porphyrin characteristic peaks, i.e., peak at − 2.5 ppm, disappeared entirely. This observation may be due to the replacement of pyrrole protons by Cu ions (the metal catalyst residue). To verify this idea, the final polymer was placed in a solution of trifluoroacetic acid in DMSO solvent at room temperature for 6 h to remove the residual ions. Purification was applied via dialysis against distilled water by using a dialysis bag with MWCO of 12 kDa. After lyophilization, the sample was analyzed again by ^1^H-NMR spectroscopy. The reappearance of pyrrole NH protons at − 2.5 ppm emphasizes the Cu ion-proton exchange process (Figure S20, inset Figure).

The number average molecular weight ($\overline{{M}_{n}})$ and polydispersity index (PDI) of Dex, Azo-grafted Dex-Br, and b-(Azo-grafted Dex)-b((MMA)-r-(β-CDAc)-r-(PorAc)) were measured by GPC, as displayed in Table S1. According to the GPC results, Azo pendant groups conjugated to the side chain of the Dex polymer were assessed as ca. 11 groups per chain. These results were in good agreement with the ^1^H-NMR analysis of Azo-grafted Dex, which showed ca. 9 groups per chain.

The mass or volume fraction of the hydrophilic block (*f*: the ratio of hydrophilic to total mass) is an important factor that influences the shape of the self-assembled block copolymers (e.g. wedges when *f* < 0.25, cylinders when *f* = 0.35 ± 0.10, and cones when *f* > 0.45). These structures are critical in determining the morphology of copolymer self-assemblies, such as worm-like micelles, spherical micelles, or polymersomes^15,32,33^. The dextran fraction (*f*_Dex_) for b-(Azo-grafted Dex)-b((MMA)-r-(β-CDAc)-r-(PorAc)) was calculated and found to be around 33%. It is worth pointing out that only the molecules with 25 < *f* < 45 would be expected to form the polymersome.

### Formation of polymersome

**Figure 2:**
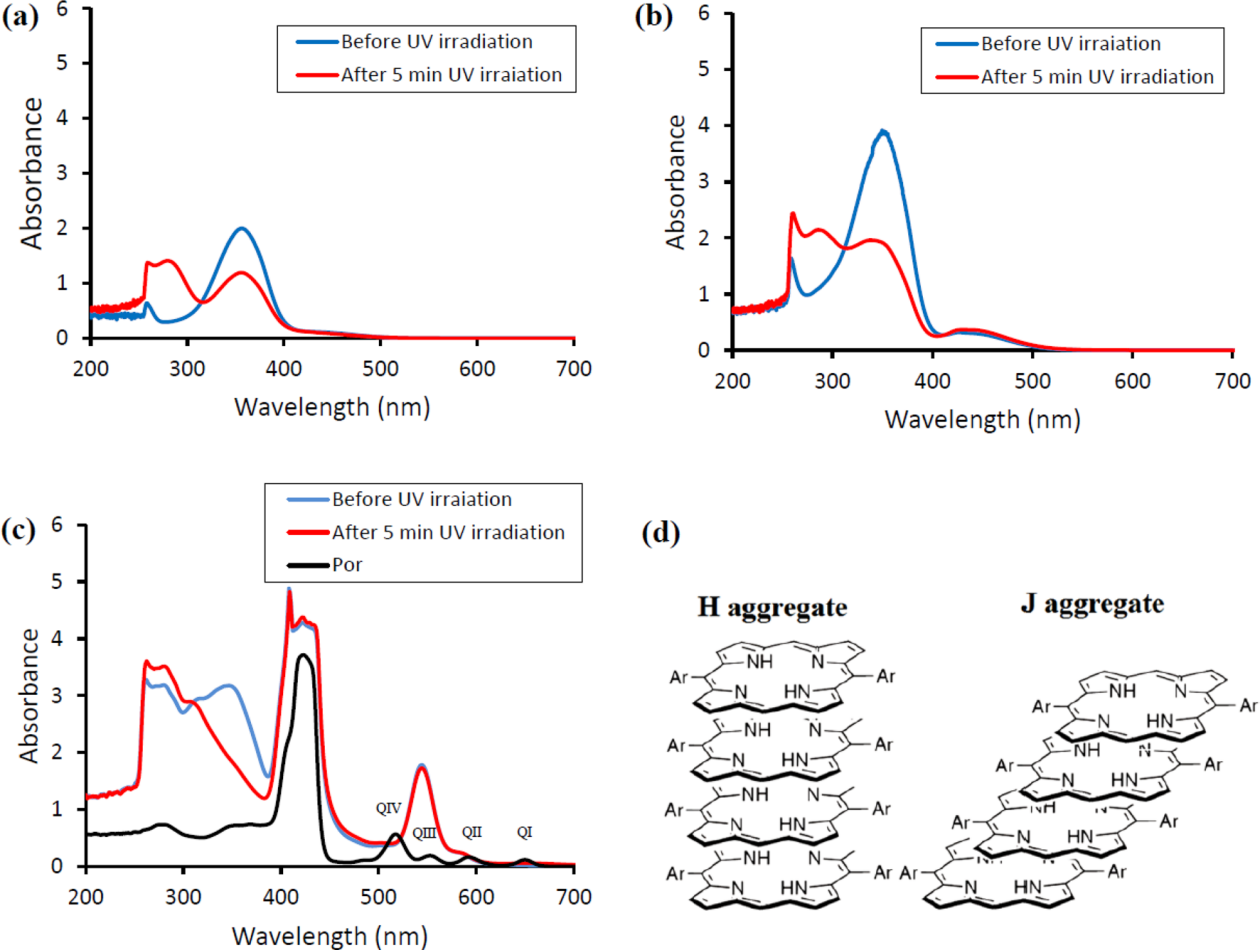
Normalized absorption spectra of Azo-OH (**a**), Macroinitiator (**b**), PorAc (black line), and polymersome before (blue line), and after (red line) UV irradiation (**c**), and Schematic illustration of H-type and J-type aggregate (**d**).

To study the structure of the aggregation state of the porphyrin in DMSO, UV–Vis spectroscopy was used. Figure 2a,b exhibit the UV–Vis spectra of Azo-OH and the macroinitiator solution in DMSO in the presence (red line) and absence (blue line) of UV irradiation (wavelength λ = 365 nm) for 5 min, respectively. The strong absorption band at ~ 360 nm belongs to the π → π* transition, while the weak peak emerged at ~ 450 nm corresponds to the n → π* transition (Fig. 2a, blue line). A drastic change was observed in the intensity ratio of the *cis*-Azo-OH compound after 5 min UV irradiation (red line). Accordingly, it is obvious that the intensity ratio of the peaks appeared at 260 and 365 nm has been changed, which reveals that a *trans–cis* isomerization in Azo-OH groups has occurred (Fig. 2a). As shown in Fig. 2b, the UV–Vis spectrum of the macroinitiator in the presence (red line) and absence (blue line) of UV irradiation shows a similar pattern with the trans–cis Azo-OH isomerization spectrum, but with a different intensity. As most of the neutral homopolysaccharides, such as Dex, are considered to have almost no UV–Vis absorption^34^, the bands appeared at ~ 360 nm and ~ 450 nm can be attributed to the π → π* and n → π* transitions of Azo-grafted groups in Dex, respectively.

The study was carried out on Por monomers and polymeric states of Por in the macrostructure in order to evaluate the π–π stacking interactions between Por groups. In Fig. 2c (black line), the spectrum shows the expected absorption bands for a monomeric Por (strong absorption at 422 nm in Soret band and four absorptions at 516, 552, 592, and 650 nm in Q band). The formation of π–π stacking interactions is confirmed from the shift and broadening in the Soret band and Q bands of UV–Vis spectra. Compared with the Soret band of monomeric Por, the intensity of the Soret band in polymeric Por has increased, the peak has split into two peaks, and the bathochromic-shift has occurred from 422 up to 427 nm (Fig. 2c). In the Q bands absorption region (500–670 nm), only Q IV has notably shifted with higher intensity, while other changes are not distinguishable due to their intensity decrease or bathochromic shift. It should be noted that, based on the previous detailed studies^25,26^, the bathochromic shift in the Soret band of the aggregated form is the result of the head-to-tail self-assembling, named J-aggregation (Fig. 2d). Side-by-side aggregation of porphyrin layers results in a blue shift toward 408 nm, known as sandwich-type H-aggregation. From all the above-mentioned results, it can be concluded that the self-assembled nanoparticle demonstrates simultaneously both J- and H-aggregations in the self-assembled system^35,36^. In another test, UV–Vis analysis of the polymersome was carried out before and after irradiating UV. It was demonstrated that no significant spectral changes occurred in the Soret band, but the *trans* to *cis* photoisomerization of the Azo was distinguishable. This suggested that the association of Por through π–π stacking was maintained unchanged even after external UV irradiation (Fig. 2c). Based on these findings, supramolecular assemblies can be entirely formed and remained stable under UV irradiation through the porphyrin conjugates formation.

### Host–guest supramolecular properties of b-(Azo-grafted Dex)-b((MMA)-r-(β-CDAc)-r-(PorAc))

**Figure 3:**
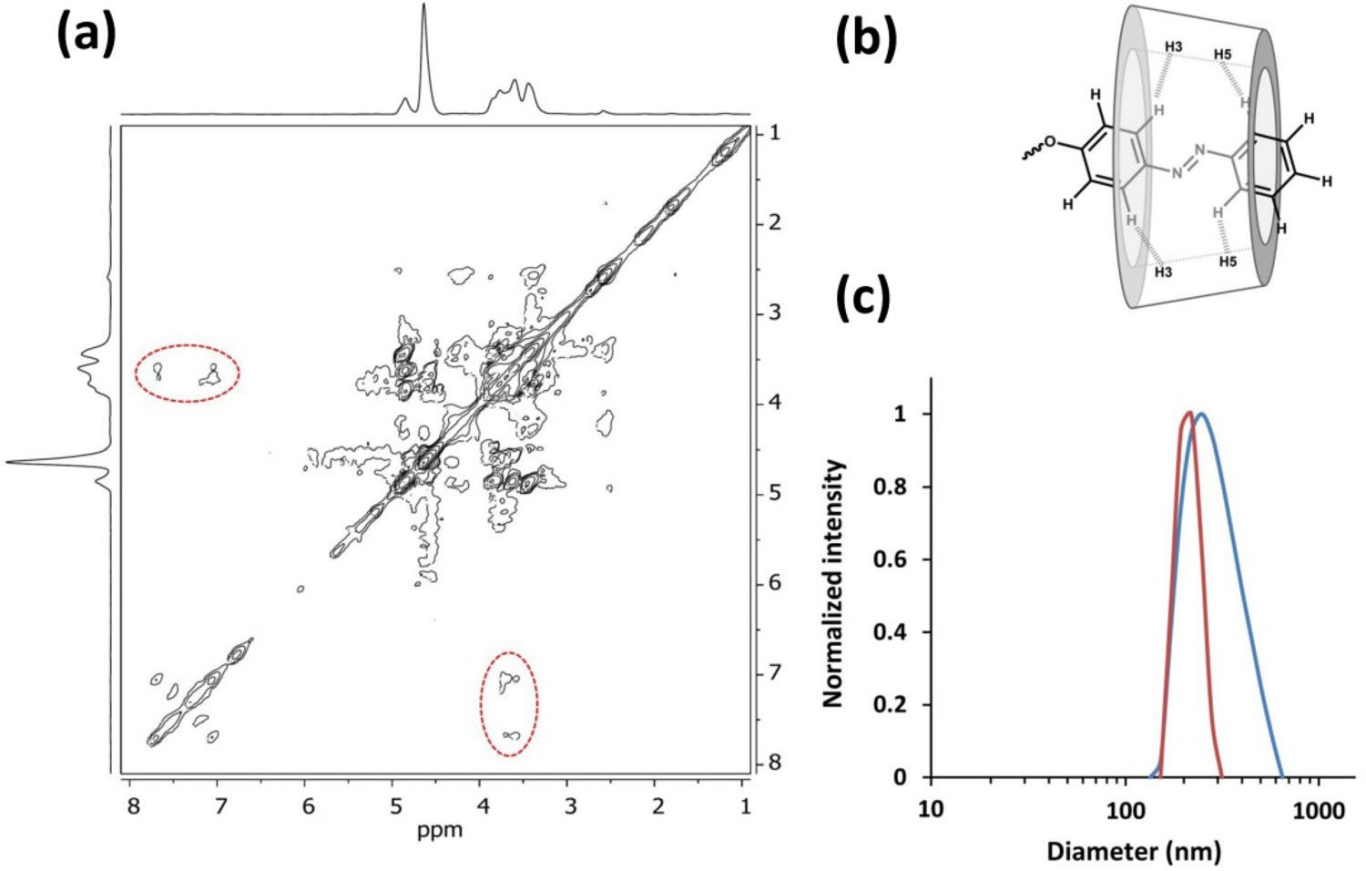
Denoised 2D NOESY NMR spectrum of b-(Azo-grafted Dex)-b((MMA)-r-(β-CDAc)-r-(PorAc)) at 25 °C in D_2_O (**a**), possible structure of the host–guest supramolecular interactions (**b**) and DLS size distribution for polymersome prior to (red) and after (blue) UV irradiation (**c**).

The host–guest supramolecular interactions of b-(Azo-grafted Dex)-b((MMA)-r-(β-CDAc)-r-(PorAc)) were characterized by 2D NOESY NMR and DLS analyses. It is worth mentioning that 2D NOESY NMR is a useful tool to study the formation of inclusion complexes. In the 2D NOESY spectrum, the cross-correlation peaks can be assigned to the protons which are spatially close to each other (< 4 Å) ^37,38^. In this work, 2D NOESY NMR was performed to confirm the formation of the β-CD ring and the Azo inclusion complex. The cross-correlation signals between the H3 and H5 inner protons of the β-CD cavity (3.54–3.76 ppm) and the aromatic protons of Azo moiety (6.95–7.73 ppm) have been evaluated in the 2D NOESY spectrum (Fig. 3a,b). It is obvious in the 2D NOESY spectrum that the signals are detected at the corresponding intersections which are expected for an inclusion complex of β-CD and Azo moieties (red dash line). Thus, close proximity between the protons of the Azo and β-CD moieties can be observed, confirming the formation of an inclusion complex. Note that the 2D NMR experiment was carried out for investigating the copolymer, partially dissolved in D_2_O during 19 elapsed hours.

To investigate the scission effect of host–guest interactions in the polymersome, DLS experiments were carried out (Fig. 3c). The obtained data indicated that there were no significant changes in the mean size of the polymersome before and after UV irradiation. Hence, this UV dependent scission effect is totally reversible, as the supramolecular β-CD/Azo host–guest complex assembles to its basic structure after leaving the polymer solution under visible light for 6 h.

The above results showed that the main architecture of the polymersome remained unchanged by breaking the host–guest interactions between β-CD and Azo. The π–π stacking aromatic interactions of Por moiety led to a high rigidity that offered structure stability even after applying UV–Vis irradiation for 5 min. Although the isomerization of the Azo units from the *trans* to *cis* form after UV irradiation caused an extent increase in the size of polymersomes, H- and J-aggregations of Por groups made polymersomes reversible structures in the absence of UV–Vis irradiation.

**Figure 4:**
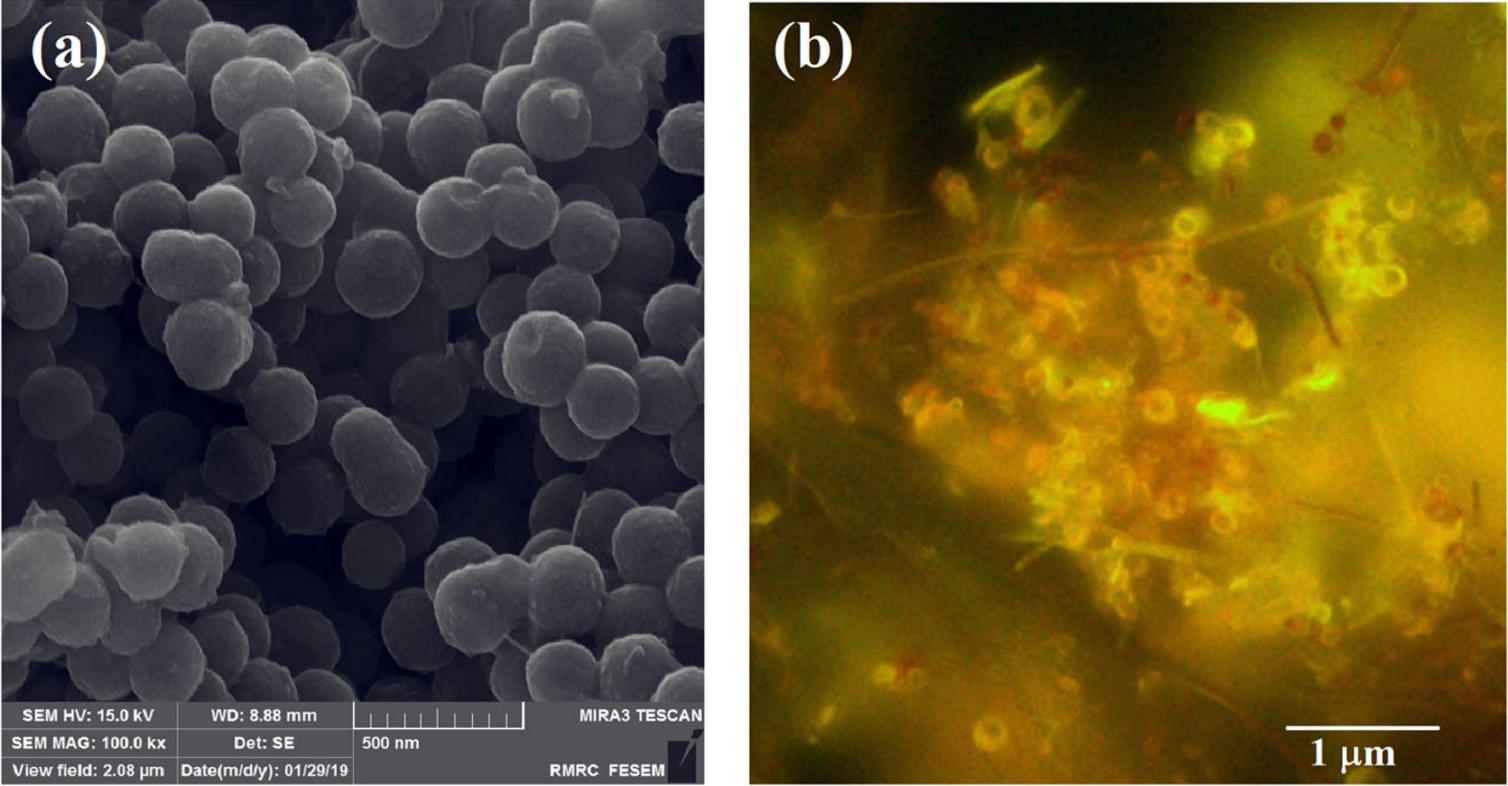
FESEM (**a**) and fluorescence microscopy (**b**) images of polymersome.

Next, to study the aggregating nature of the polymersome, FESEM and Fluorescence Microscopy were used. As can be seen in the FESEM images, spherical polymersomes have a diameter of about 200 nm (Fig. 4a). Fluorescence microscopy results are in accordance with the FESEM ones, confirming the vesicular morphology arising from the presence of shining golden rings, scattered from Azo groups, as fluorescent agent, in the corona part of polymersomes (Fig. 4b).

**Figure 5:**
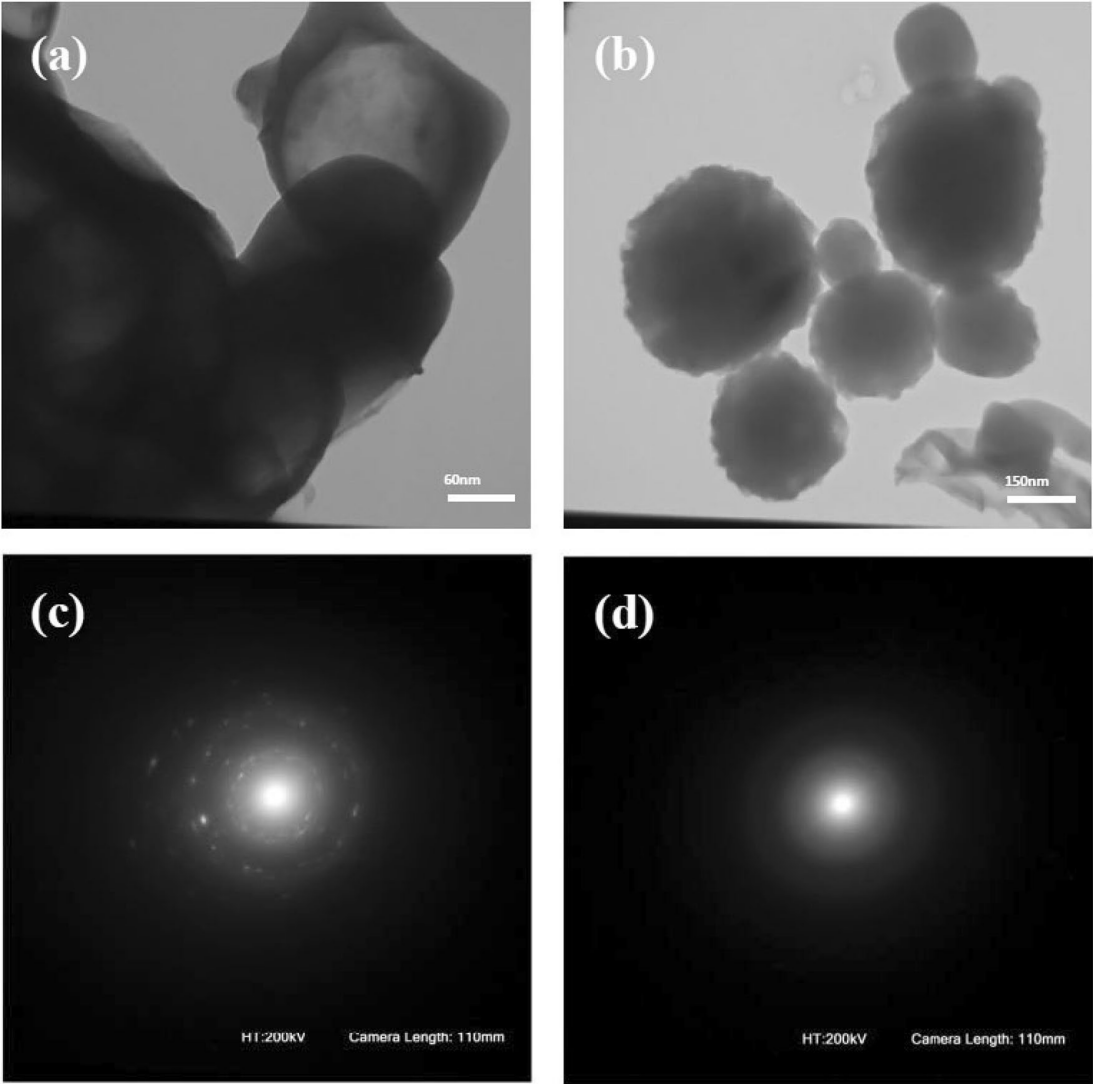
TEM images of polymersomes before (**a**) and after (**b**) UV irradiation and their corresponding SAED patterns (**c**,**d**).

We also studied visualizing the aggregates formed in the morphology by using Transmission Electron Microscopy (TEM). TEM images of the amphiphilic copolymers were obtained by investigating a 0.2 mg mL^−1^ THF solution (Fig. 5).

Based on the TEM and DLS results, the self-assembly of particles before and after UV irradiation is remained intact but with some slight variations in the shape of the nanoparticles. In the absence of UV irradiation, particles were formed through host–guest supramolecular interactions, which were self-assembled into vesicle-like structures with an average diameter of ∼ 200 nm (Fig. 5a) and some aggregations, while in the presence of UV irradiation, bigger particles with a mean diameter of ∼ 250–300 nm and much less aggregations were observed (Fig. 5b). Therefore, when the polymersome solution was exposed to UV light, the *trans*-Azo was transformed into the *cis*-Azo form, leading to dissociation of the Azo from the β-CD cavity, and the intermolecular supramolecular polymers were destroyed. Also, the *cis*-Azo occupied a bigger volume and formed a more polar system than the *trans*-structure. This larger volume is needed for the *cis*-form to exist in the stable state^39^. Thus, we observed bigger and more separated particles in TEM and DLS after UV irradiation.

On the other hand, as exhibited in Fig. 5a, before UV irradiation, the nanoparticles show a clear contrast between the center and the periphery, and the surface of the particles is smooth, which is due to the typical vesicular structures of these types of vehicles. However, after UV irradiation, by dissociation of the host–guest supramolecular structure, the orientation of polymer chains was destroyed and the chains expanded. So, in Fig. 5b, we can observe the particles that do not have smooth surfaces in both the outer and inner surfaces, while the main architecture of these particles has remained unchanged. Selected-area electron diffraction (SAED) analysis was used to further verify the above results. As shown in Fig. 5c,d, SAED patterns indicate that, before UV irradiation, particles exhibit small spots which form a ring, demonstrating a polycrystalline nature, while it is not observed after UV irradiation.

**Figure 6:**
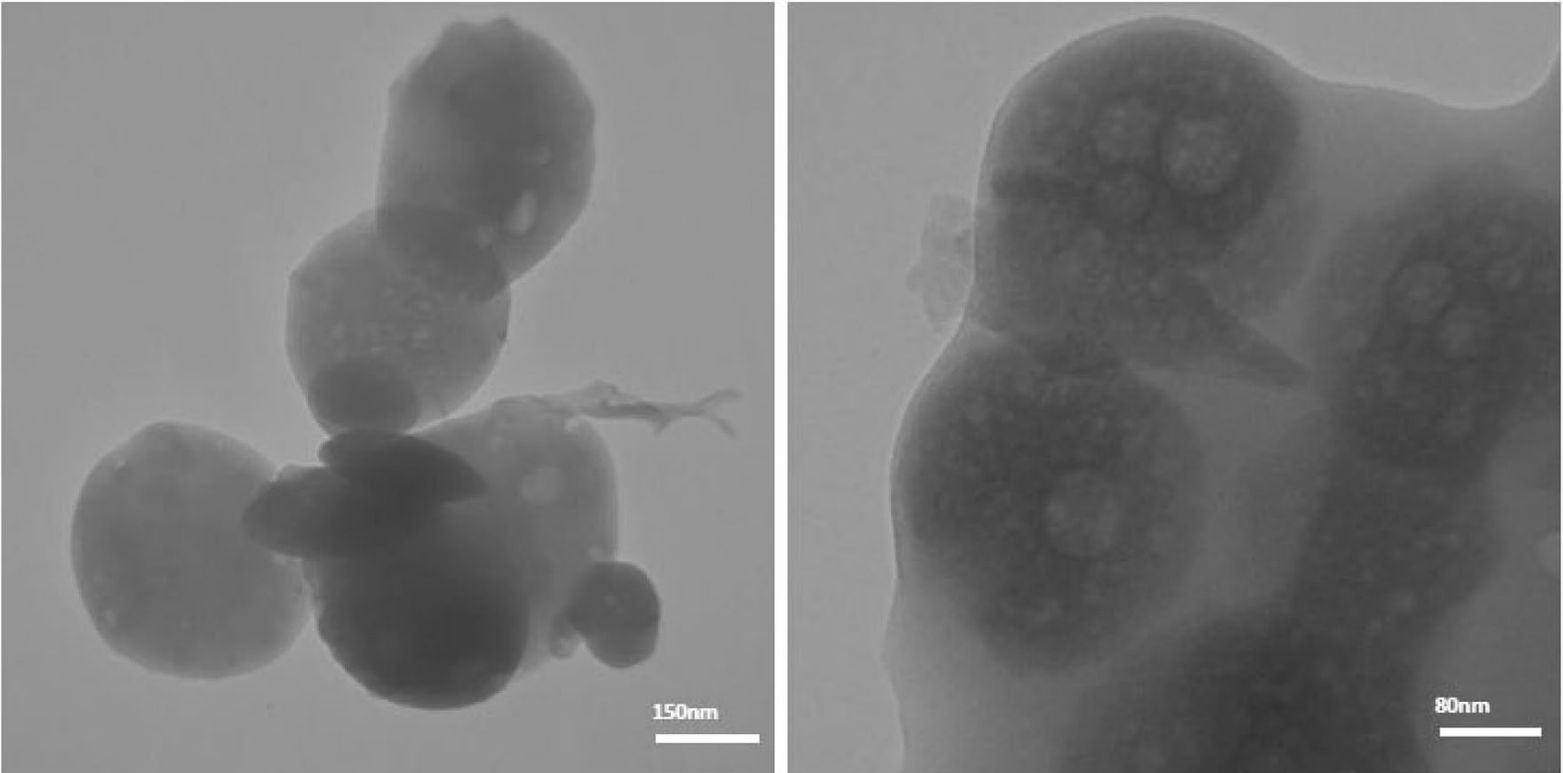
TEM images of polymersomes after 30 min under visible-light irradiation.

Then, the ability of the aggregate transition to be reversed by visible-light irradiation was determined based on the reversible isomerization of the Azo moiety. For this purpose, the polymersomes were exposed to visible-light irradiation for 30 min and TEM analysis was performed again to confirm this phenomenon. Fortunately, as depicted in Fig. 6, we successfully captured the transition state by TEM analysis, clearly showing the embryonic vesicles with stained corona. These properties suggest that these particles behave as a good candidate for use as photo-controlled reversible particles for on-demand release of drugs.

### PDT properties of polymersome

**Figure 7:**
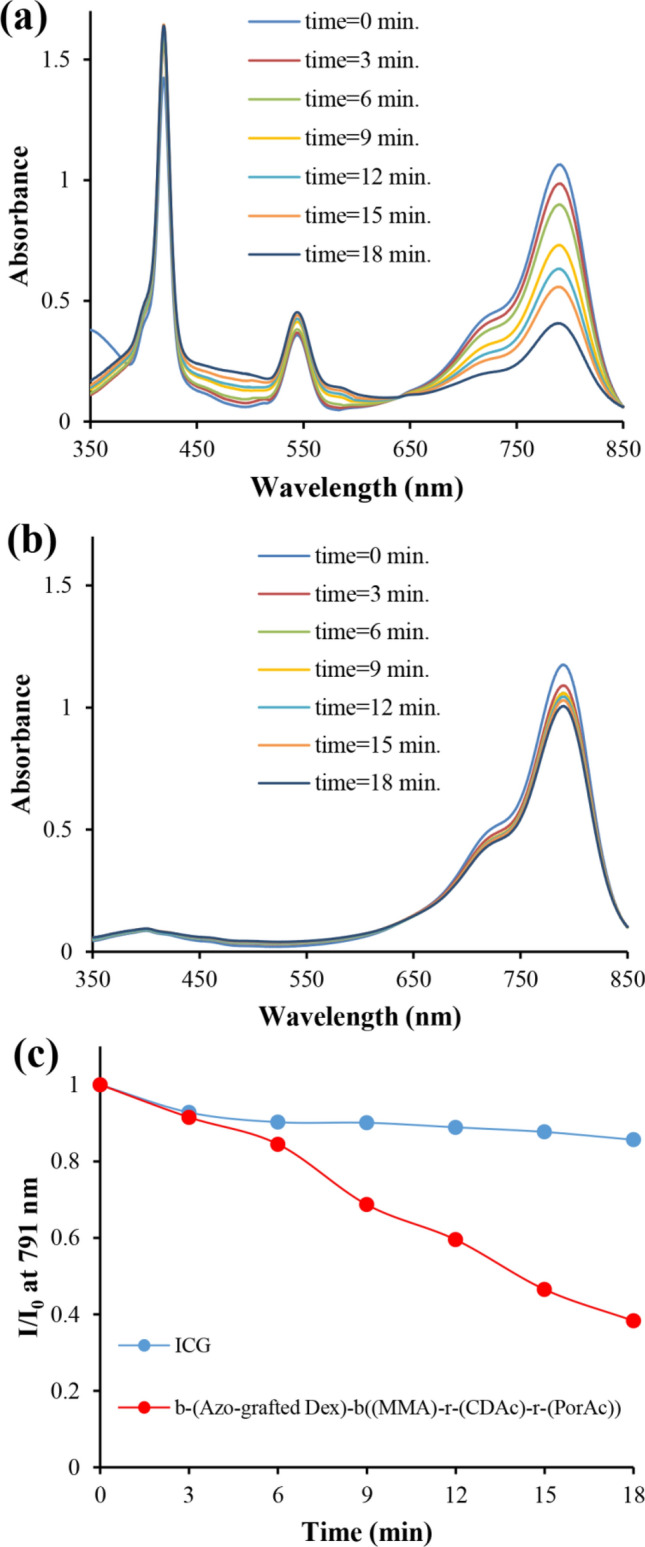
Time-dependent absorption spectra of (**a**) ICG in the presence of polymersome; (**b**) ICG alone; (**c**) decomposition rate of ICG alone (Blue) and ICG in the presence of polymersome under irradiation (Red).

The photodynamic properties of the polymersome in the production of singlet oxygen were also studied. Indocyanine green (ICG) was used as an indicator to evaluate the Por ability to generate singlet oxygen under photo-irradiation, which was monitored by time-dependent absorption spectroscopy. The UV absorption spectra of ICG in the presence of polymersomes (0.1 mg/mL) and ICG blank (1 mg/mL) in a DMF solution were measured under irradiation (Fig. 7). Sampling was done every three minutes. As shown in Fig. 7b, the irradiated ICG solution in DMF, as a control experiment, only shows a negligible decrease in the absorbance of 791 nm (the maximum absorption of ICG in DMF). The absorbance of ICG in the presence of polymersomes gradually decreases as the extension of irradiation time, indicating that Por molecules can result in a steady generation of singlet oxygen under irradiation (Fig. 7a). For a photosensitive molecule, photostability is an important feature to achieve photodynamic therapy. The absorption intensity in ~ 425 nm and ~ 540 nm, which belongs to the particles, shows negligible changes, indicating that the Por moiety conjugates possess a good photostability. As a comparison between decomposition rates of ICG in the presence and the absence of the particles under irradiation, the relative ratios of the ICG absorbance at 791 nm with the initial value and various time values were plotted as a function of the irradiation time (Fig. 7c). The absorbance of ICG decreased in the presence of polymersomes at 791 nm and reached 38% under irradiation, while the absorbance of the blank ICG was maintained at 86%, indicating the effective generation of singlet oxygen of Por moiety in the particles.

According to the literature, the large π-conjugates of porphyrin molecules often undergo closely packed aggregation, such as H aggregation, which decreases singlet oxygen generation due to self-quenching of the excited state, greatly limiting the application of these molecules in PDT application^25,26,40^. Thus, the steady generation of singlet oxygen under irradiation strongly proves the presence of J-aggregation in polymersomes.

### Release behavior of polymersome

**Figure 8:**
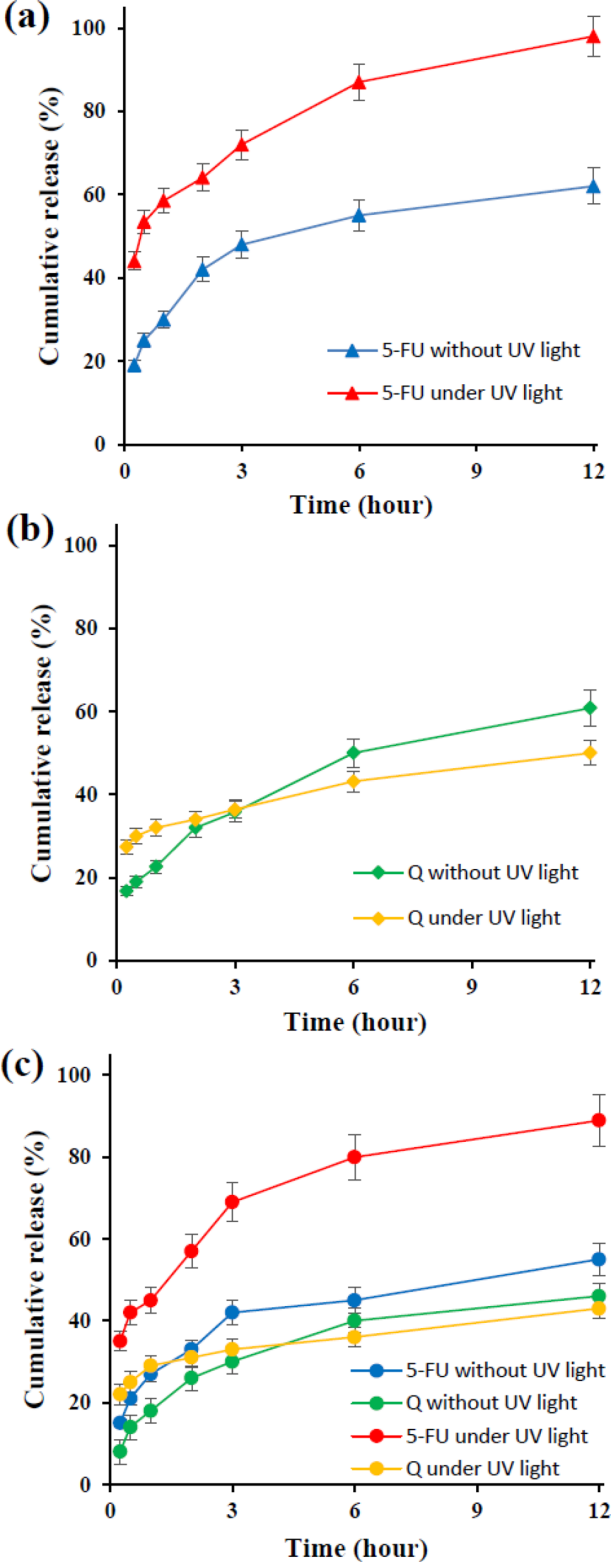
The cumulative drug release behavior of polymersomes at physiological temperature and pH = 7.4, 5-FU drug release behavior under UV light (red) and without UV light (blue) (**a**) Q drug release behavior under UV light (yellow) and without UV light (green) (**b**) mixed drug release profiles: 5-FU release profiles under (red) and without (blue) UV light irradiation, Q release profiles under UV light (yellow) and without UV light (green) irradiation (**c**).

Loading the anti-cancer drugs provides a significant opportunity for the design of advanced single/dual-drug loading nanocarriers. Quercetin (Q) and 5-Fluorouracil (5-FU) molecules were used as the model therapeutic agents to investigate the in vitro drug delivery of prepared polymersome. Release behavior of Q and 5-FU was previously studied and it was demonstrated that it occurred in different manners due to the different conditions^41^. Here, the study was performed for Q, 5-FU, and the mixed drug system, in the presence and absence of UV irradiation (365 nm) to indicate the role of the supramolecular scission effect on drug release. The drug loading content and encapsulation efficiency of Q, 5-FU, and dual-drug loaded nanoparticles are listed in Table S2. In all samples and conditions (Fig. 8a–c), the 5-FU release was higher than that of Q due to its hydrophilic character. Azo containing parts of the copolymer reversibly undergo *cis* and *trans* isomerization under UV or visible conditions. Just trans-Azo configuring can make host–guest structure with β-CD in normal conditions. The scission of host–guest interactions under UV irradiation leads to a faster release behavior of 5-FU when compared to the situation without applying irradiation, but Q release behavior is different. As shown in Fig. 8b, after the first 3 h of drug release, the slope of the Q release curve decreases slightly in the presence of UV irradiation, and it exhibits a slower release behavior in comparison with that occurs in the absence of UV irradiation, which is due to the photodegradation of Q under UV light^42,43^. In the mixed drug loading system (Fig. 8c), the Q release curve shows less variation during UV irradiation, which may be due to the physical interactions of the two drugs in the release media, which leads to further decreasing in photodegradation of Q under UV light. To show this effect, the interactions of 5FU, Q, and the mixture of these two drugs were investigated by using UV–Vis spectroscopy in DMSO solvent and release media (Fig. S21). As shown in Fig. S21a, the UV–Vis spectrum of the drugs mixture exhibits the characteristic absorption peaks of both drugs simultaneously without any significant shifts, which indicates the absence of any drug-drug interactions in DMSO. According to Fig. S21b, the spectra of Q and 5-FU exhibits absorption peaks at 215 and 275 nm, respectively, while the spectrum of the two drugs mixture shows absorption peaks at 202 and 240 nm. It was found that the UV–Vis spectra of the two drugs were different before and after their mixing, indicating that a drug-drug interaction had occurred in the PBS solution.

## Conclusion

In the present study, we have constructed an amphiphilic block copolymer, b-(Azo-grafted Dex)-b((MMA)-r-(β-CDAc)-r-(PorAc)) via ATRP polymerization of predesigned β-CD and Por acrylate monomers from the hydrophilic Azo-grafted Dex-Br macroinitiator. The amphiphilic copolymers were self-assembled into polymersomes in water. The Por units effectively formed H- and J-aggregations within the polymeric layer, where these aggregates reduced aggregation-induced quenching and acted as non-covalent cross-linking points. Due to the rigidity of Por-Por interactions (H- and J-aggregate) by incorporation of β-CD and Azo via host–guest interactions, a reversible stimuli-responsive polymersome was prepared. The results of DLS, TEM, FESEM, and fluorescence microscopy showed the vesicular morphology and confirmed the reversibility characteristics of the particles. The unique π–π stacking interaction and host–guest chemistry demonstrate a superior performance of b-(Azo-grafted Dex)-b((MMA)-r-(β-CDAc)-r-(PorAc)), which can be a promising candidate for single/dual drug loading of hydrophobic and hydrophilic model drugs. This novel architecture opens a promising opportunity to reduce the aggregation-induced quenching phenomenon and improve the singlet oxygen generation capability of Por photosensitizers.

## Supplementary Information

Supplementary Information.
