# Supplementary material for: The simultaneous role of porphyrins’ H- and J- aggregates and host–guest chemistry on the fabrication of reversible Dextran-PMMA polymersome
Source: Sci Rep. 2021 Feb 2;11:2832. doi: 10.1038/s41598-021-82256-7 (PMC7854723; doi:10.1038/s41598-021-82256-7)
Supplement: Supplementary file 1 — Supplementary Information. [file 41598_2021_82256_MOESM1_ESM.pdf]

Supplementary Information

The simultaneous role of porphyrins' H- and J- aggregates and host-guest chemistry on the fabrication of reversible Dextran-PMMA polymersome

Seyed Milad Safar Sajadi * and Sepideh Khoee *

Polymer Laboratory, School of Chemistry, College of Science, University of Tehran, Tehran, Iran. PO Box 14155 6455.

* Corresponding authors, Email: m.safarsajadi@ut.ac.ir (S. M. Safar Sajadi)

Email: Khoee@Khayam.ut.ac.ir (S. Khoee)

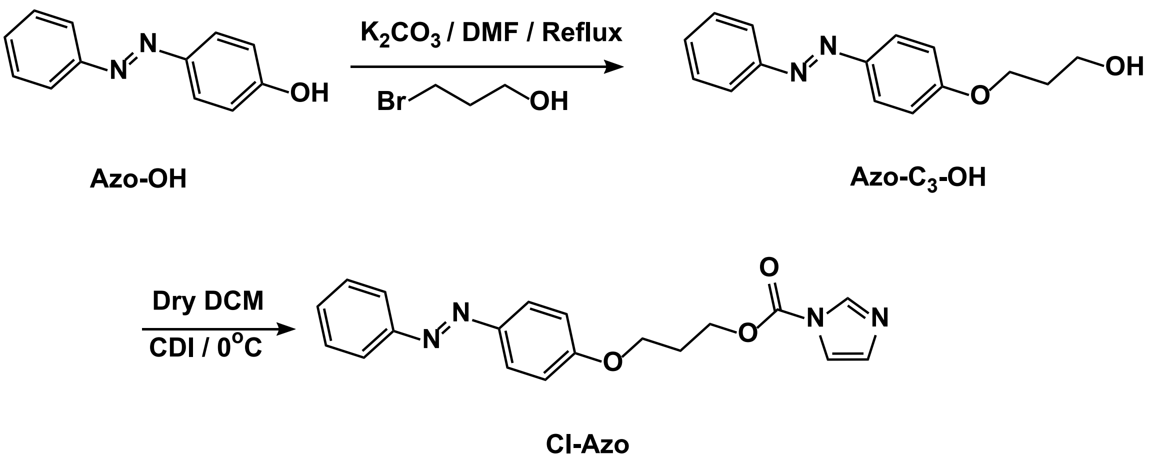

Figure S1. Synthesis of CI-Azo

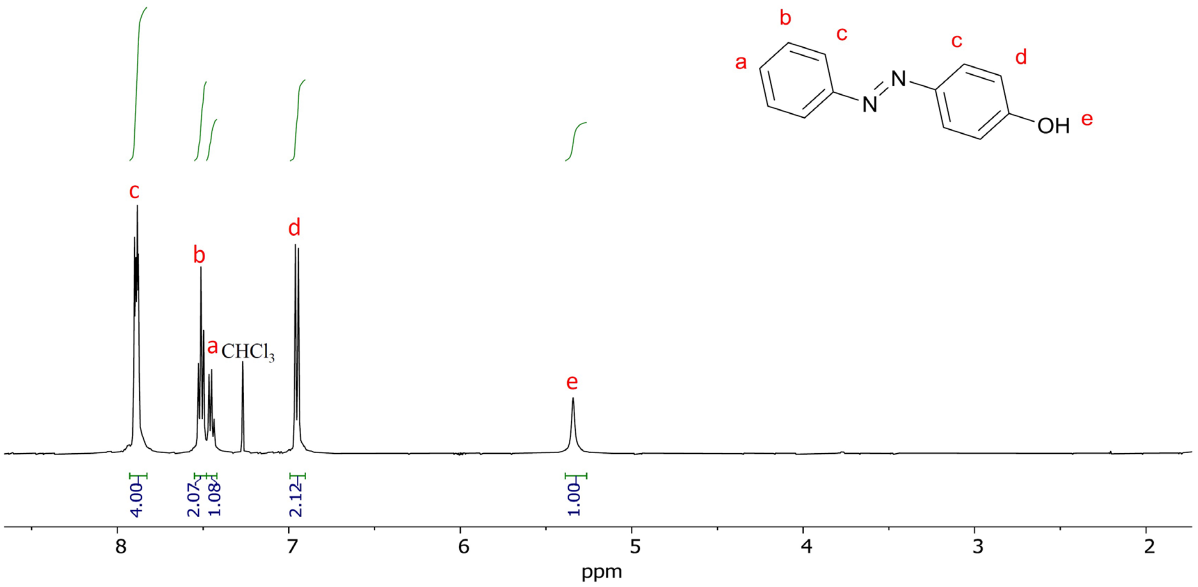

Figure S2. ^{1}H-NMR spectrum of Azo-OH in $CDCl_3$.

^{1}H-NMR (500 MHz, $CDCl_3$): δ (ppm) 5.34 (s, 1H), 6.95 (d, 2H), 7.44 (t, 1H), 7.51 (t, 2H), 7.88 (m, 4H).

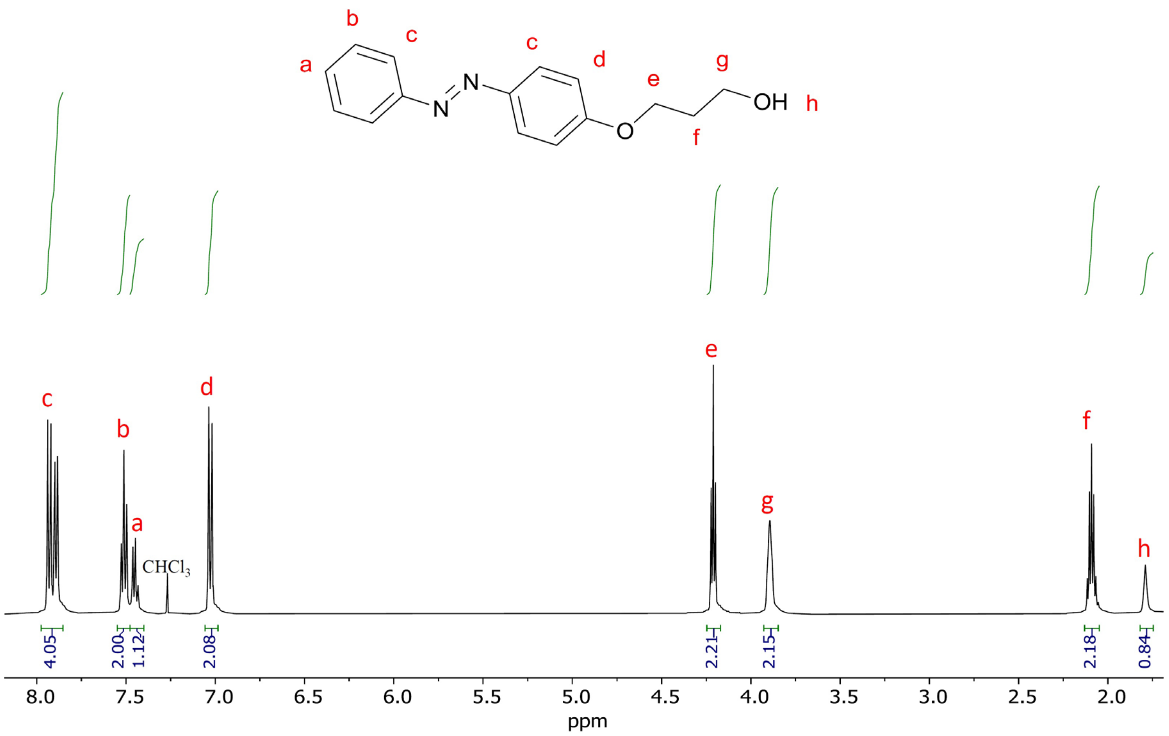

Figure S3. ^{1}H-NMR spectrum of Azo-C$_3$-OH in CDCl$_3$.

^{1}H-NMR (500 MHz, $CDCl_3$): δ (ppm) 1.78 (s, 1H), 2.09 (q, 2H), 3.89 (t, 2H), 4.21 (t, 2H), 7.02 (d, 2H), 7.44 (t, 1H), 7.51 (t, 2H) 7.90 (q, 4H).

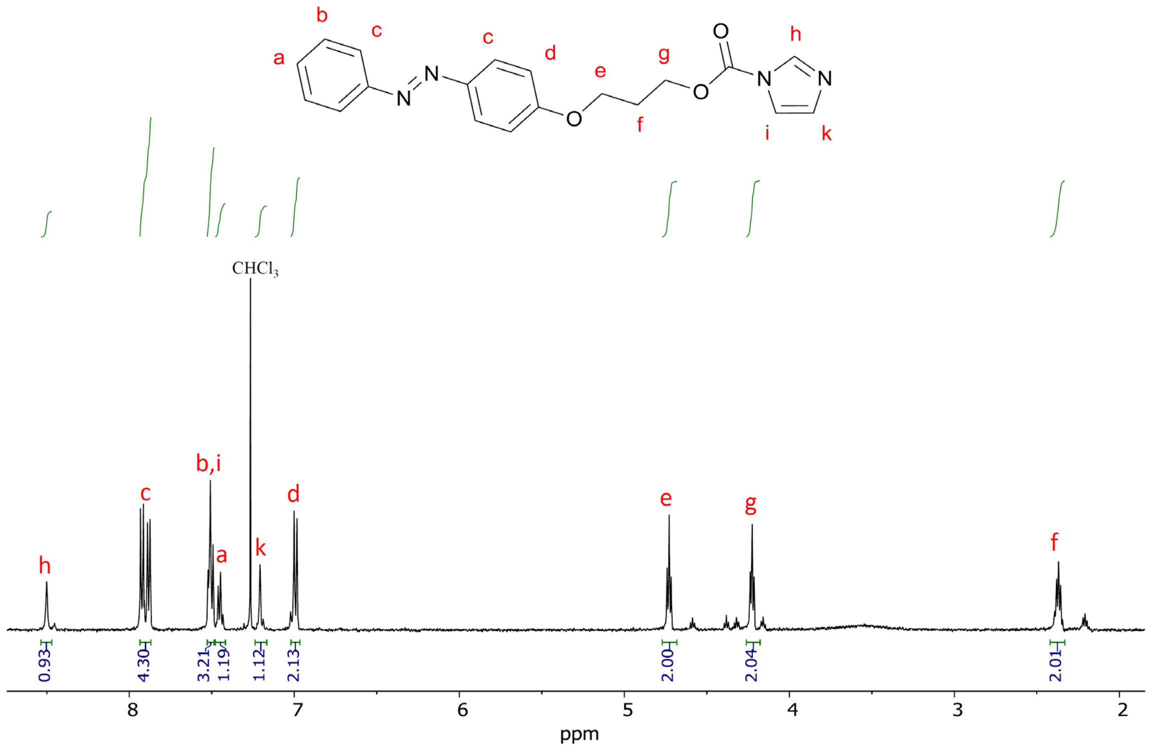

Figure S4. ¹H-NMR spectrum of CI-Azo in $CDCl_3$.

^{1}H-NMR (500 MHz, $CDCl_3$): δ (ppm) 2.36 (q, 2H), 4.22 (t, 2H), 4.72 (t, 2H), 6.99 (d, 2H), 7.20 (d, 1H), 7.44 (t, 1H), 7.50 (m, 3H), 7.90 (q, 4H), 8.50 (s, 1H).

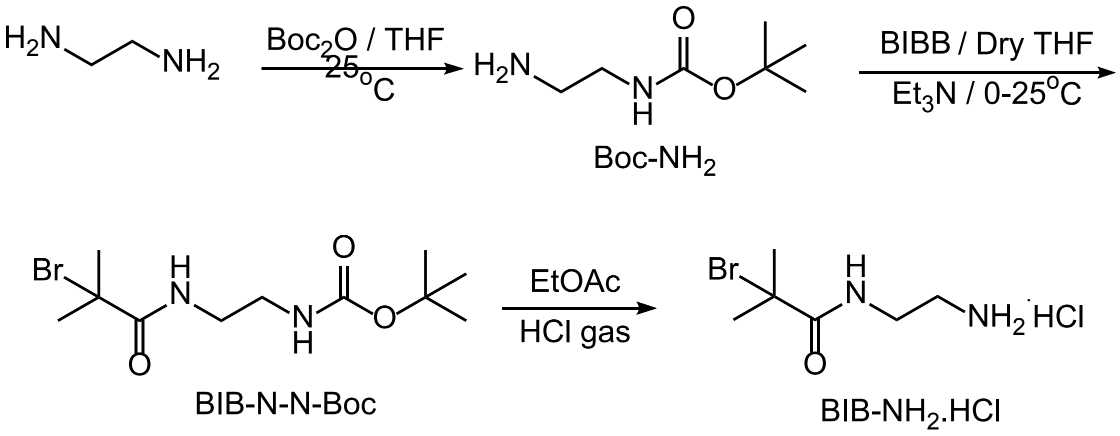

Figure S5. Synthesis of BIB-NH_2.HCl

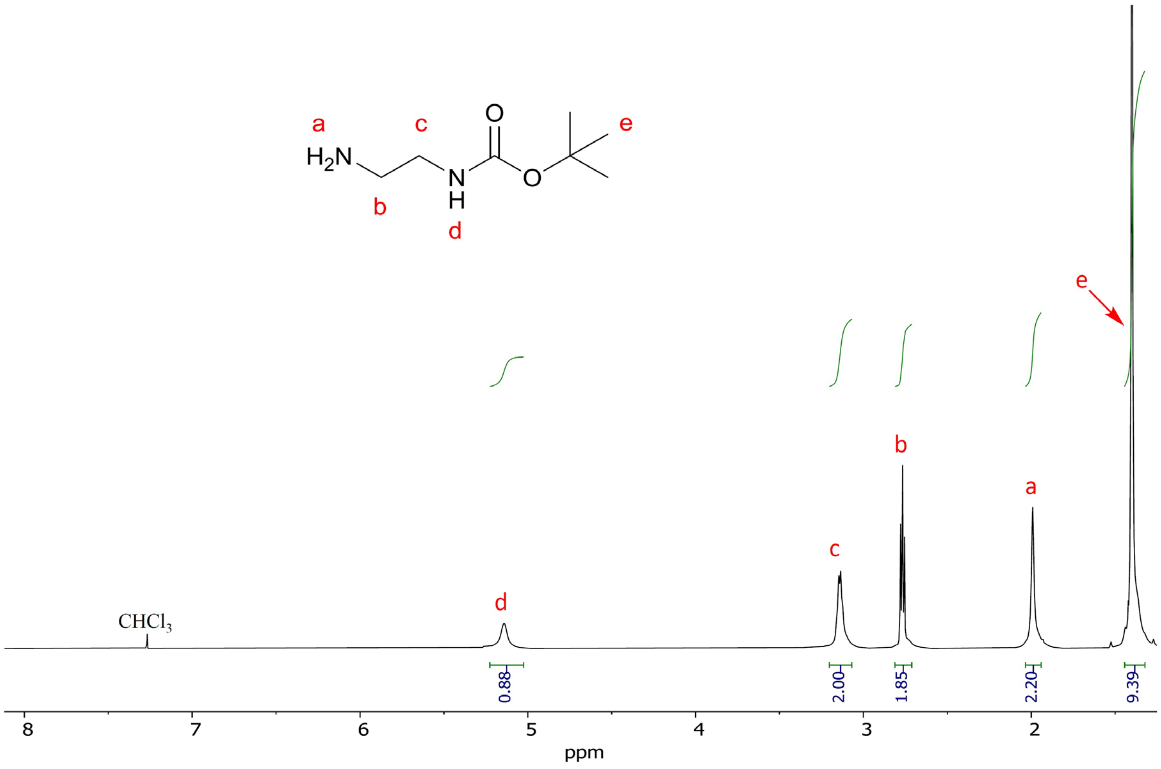

Figure S6. ¹H-NMR spectrum of Boc-NH_2 in $CDCl_3$.

^{1}H-NMR (500 MHz, $CDCl_3$): δ (ppm) 1.39 (s, 9H), 1.99 (s, 2H), 2.76 (t, 2H), 3.14 (q, 2H), 5.14 (s, 1H).

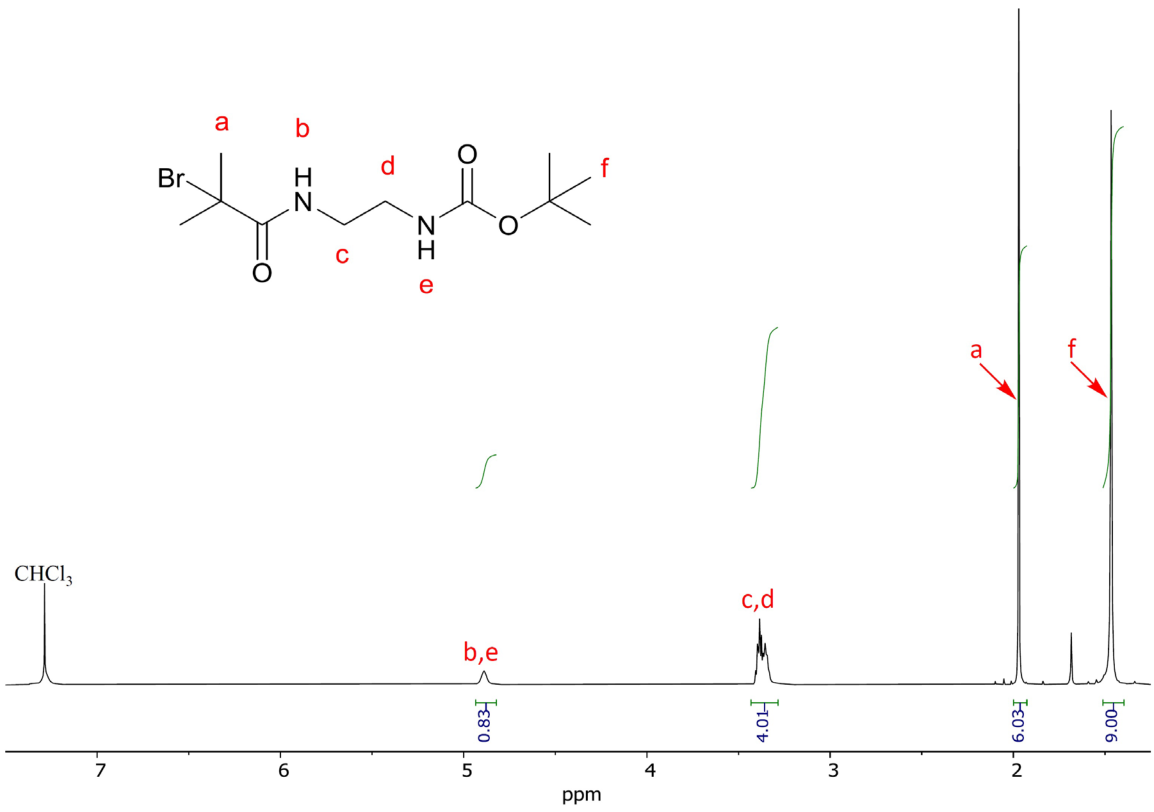

Figure S7. ^{1}**H-NMR spectrum of BIB-N-N-Boc in** $\mathbf{CDCl_3}$**.**

^{1}H-NMR (500 MHz, $CDCl_3$): δ (ppm) 1.46 (s, 9H), 1.97 (s, 6H), 3.37 (m, 4H), 4.89 (s, 2H).

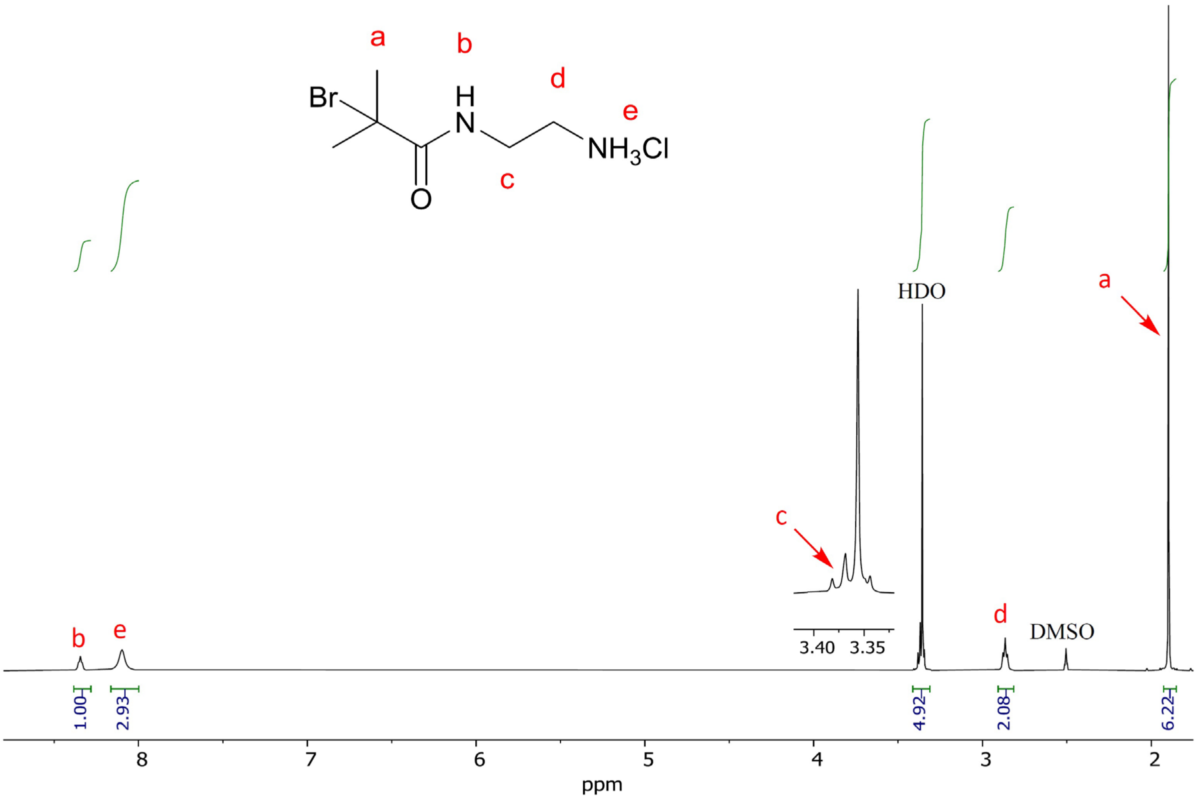

Figure S8. ^{1}H-NMR spectrum of BIB-NH_2.HCl in DMSO-d_6. (Signal in 3.37 ppm partially overlapped with HDO signal)

^{1}H-NMR (500 MHz, DMSO-d_6): δ (ppm) 1.89 (s, 6H), 2.86 (t, 2H), 3.37 (q, 2H), 8.09 (s, 3H), 8.34 (t, 1H).

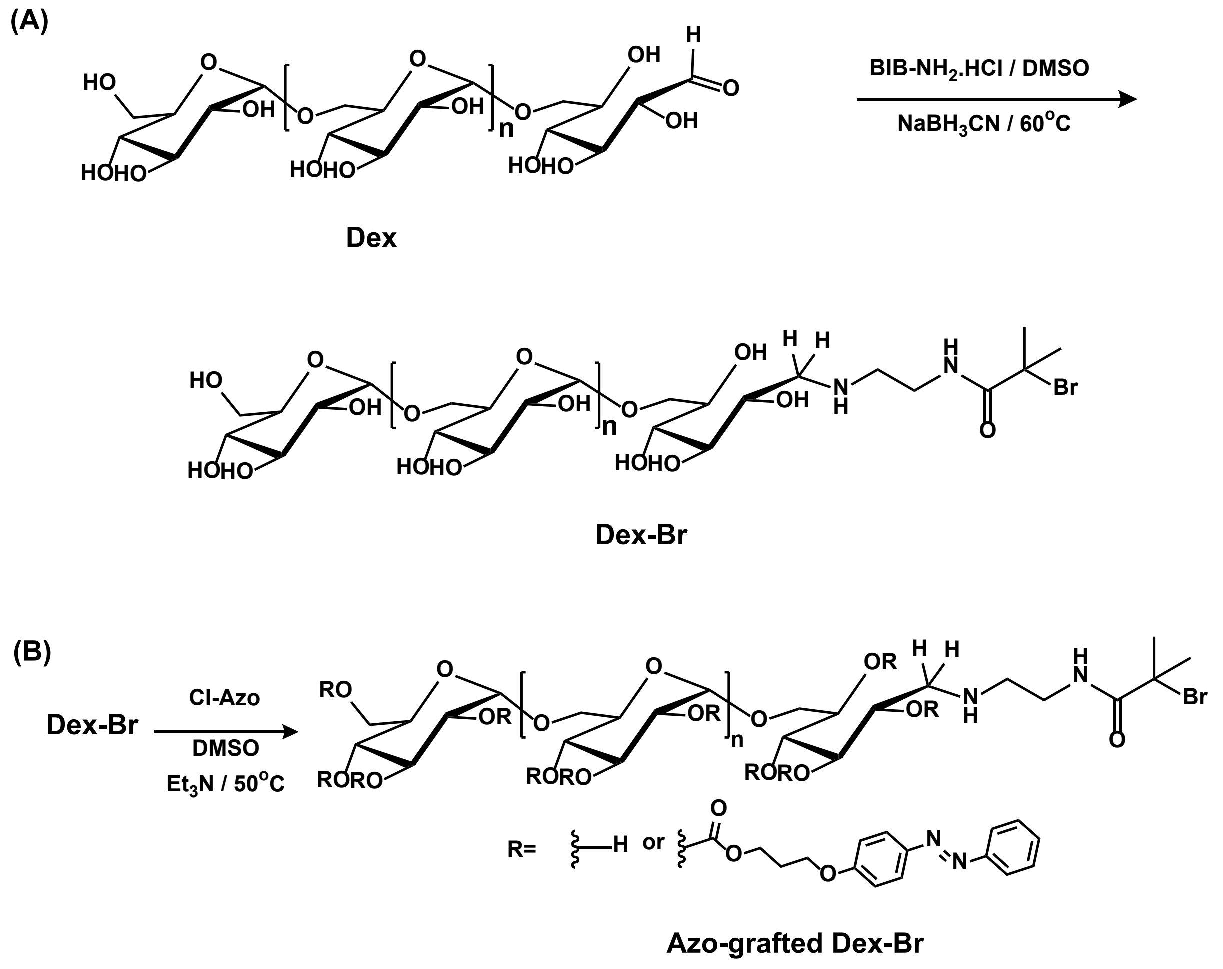

Figure S9. Synthesis of Dex-Br (a) and Azo-grafted Dex-Br (b)

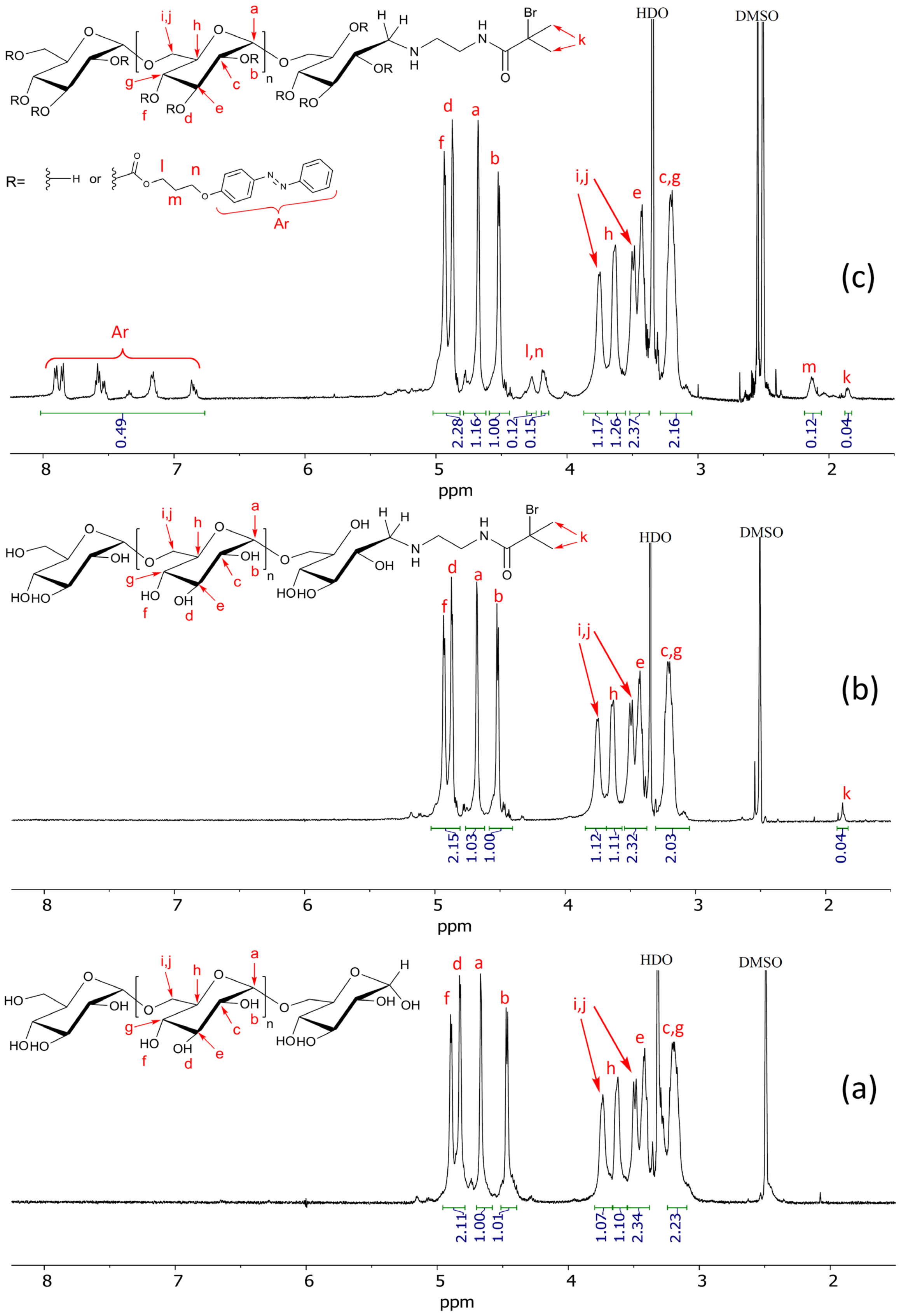

Figure S10. ^{1}H-NMR Spectra of Dex (a), Dex-Br (b), and Azo-grafted Dex-Br (c) in DMSO-d$_6$.

(a): ^{1}H-NMR (500 MHz, DMSO-d_6): δ (ppm) 3.09-3.24 (c, g), 3.37-3.54 (e, i, j), 3.55-3.65 (h), 3.66-3.79 (i, j), 4.39-4.51 (b), 4.58-4.69 (a, Anomeric H), 4.79-4.95 (d, f).

(b): ^{1}H-NMR (500 MHz, DMSO-d_6): δ (ppm) 1.87 (k, C$\boldsymbol{H}_3$), 3.04-3.30 (c, g), 3.37-3.54 (e, i, j), 3.56-3.68 (h), 3.68-3.84 (i, j), 4.40-4.58 (b), 4.61-4.76 (a, Anomeric H), 4.80-5.03 (d, f).

(c): ^{1}H-NMR (500 MHz, DMSO-d_6): δ (ppm) 1.85 (k, C$\boldsymbol{H}_3$), 2.12 (m, $CH_2C\boldsymbol{H}_2CH_2$), 3.04-3.28 (c, g), 3.37-3.52 (e, i, j), 3.55-3.66 (h), 3.66-3.87 (i, j), 4.17 (n, Ar-OC$\boldsymbol{H}_2$CH$_2$), 4.26 (l, $CH_2C\boldsymbol{H}_2$-OC=O), 4.43-4.59 (b), 4.62-4.79 (a, Anomeric H), 4.81-5.02 (d, f), 6.76-8.01 (Aromatic H).

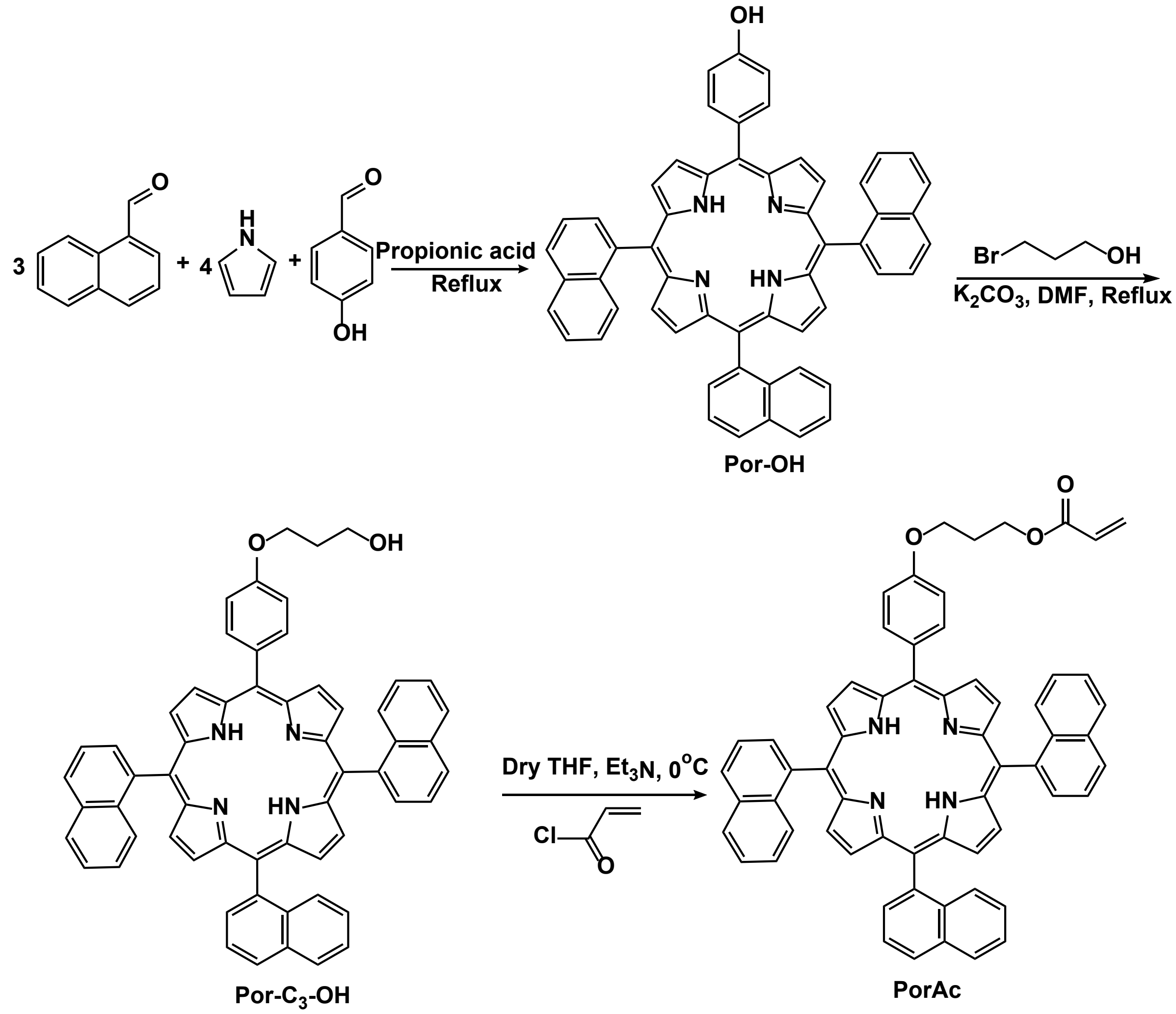

Figure S11. Synthesis route of PorAc

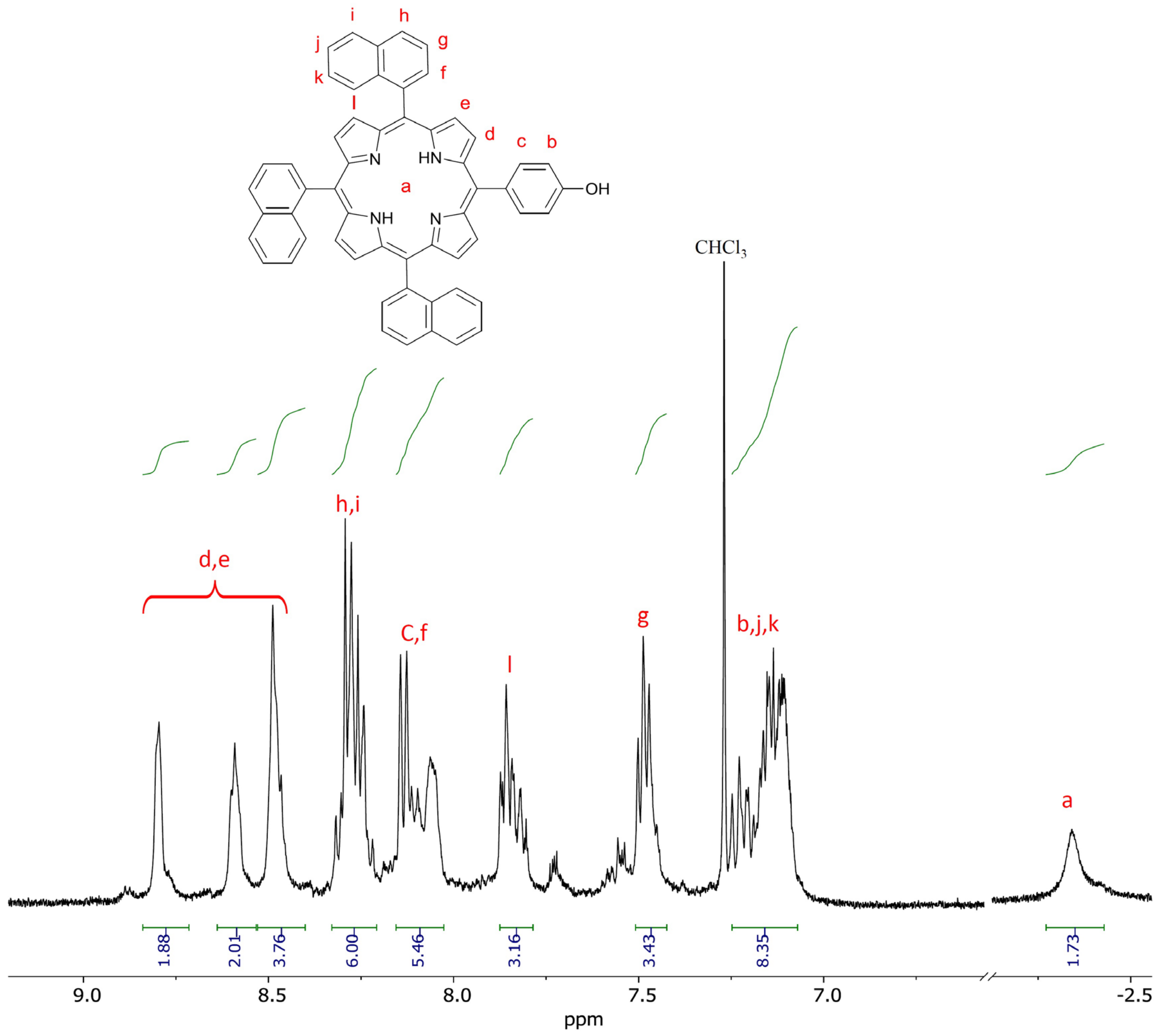

Figure S12. ^{1}**H-NMR spectrum of Por-OH in** $CDCl_3$**.**

^{1}H-NMR (500 MHz, $CDCl_3$): δ (ppm) -2.34 (s, 2H), 7.06-7.26 (m, 8H), 7.42-7.50 (m, 3H), 7.78-7.87 (m, 3H), 8.00-8.15 (m, 5H), 8.21-8.33 (m, 6H), 8.40-8.52 (m, 4H), 8.55-8.63 (m, 2H), 8.73-8.83 (m, 2H).

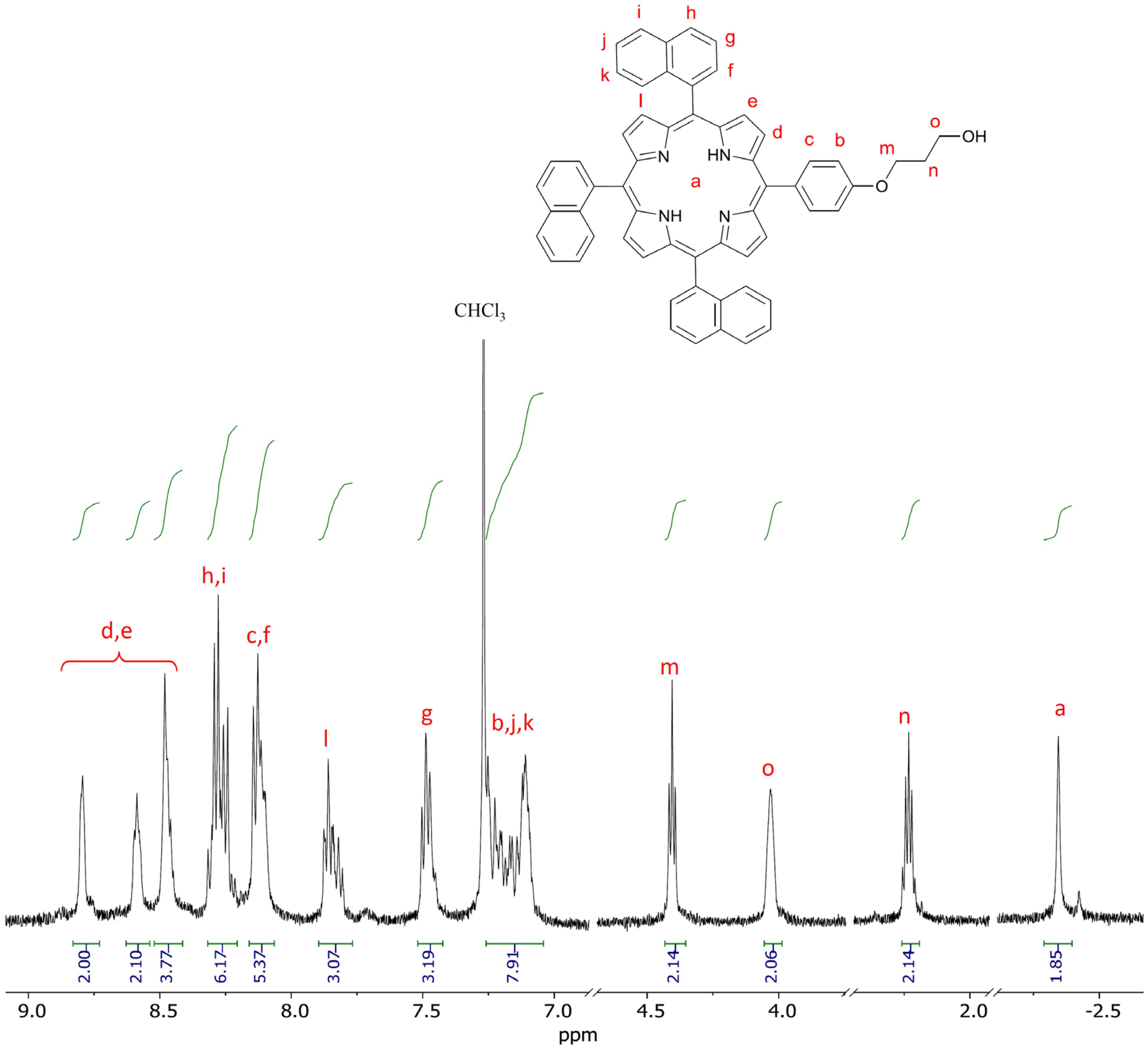

Figure S13. ^{1}**H-NMR spectrum of Por-C**$_3$**-OH in CDCl**$_3$**.**

^{1}H-NMR (500 MHz, $CDCl_3$): δ (ppm) -2.34 (s, 2H), 2.23 (q, 2H), 4.03 (t, 2H), 4.4 (t, 2H), 7.07-7.25 (m, 8H), 7.43-7.51 (m, 3H), 7.79-7.88 (m, 3H), 8.06-8.16 (m, 5H), 8.23-8.32 (m, 6H), 8.43-8.51 (m, 4H), 8.55-8.61 (m, 2H), 8.76-8.82 (m, 2H).

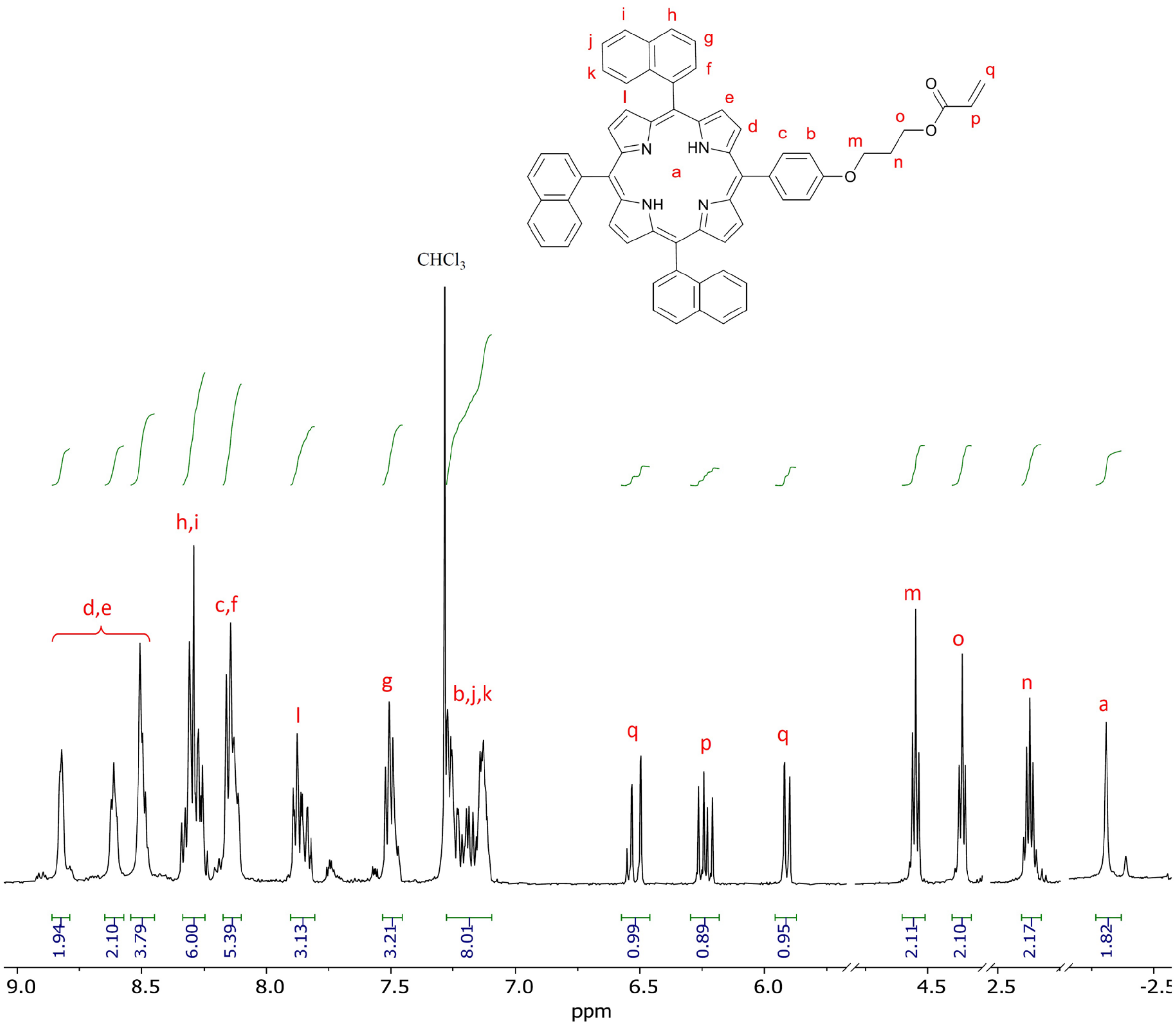

Figure S14. **^{1}H-NMR spectrum of PorAc monomer in $CDCl_3$.**

^{1}H-NMR (500 MHz, $CDCl_3$): δ (ppm) -2.30 (s, 2H), 2.37 (q, 2H), 4.36 (t, 2H), 4.54 (t, 2H), 5.90 (d, 1H), 6.23 (dd, 1H), 6.51 (d, 1H), 7.09-7.28 (m, 8H), 7.46-7.53 (m, 3H), 7.80-7.90 (m, 3H), 8.10-8.17 (m, 5H), 8.24-8.35 (m, 6H), 8.46-8.54 (m, 4H), 8.58-8.64 (m, 2H), 8.79-8.85 (m, 2H).

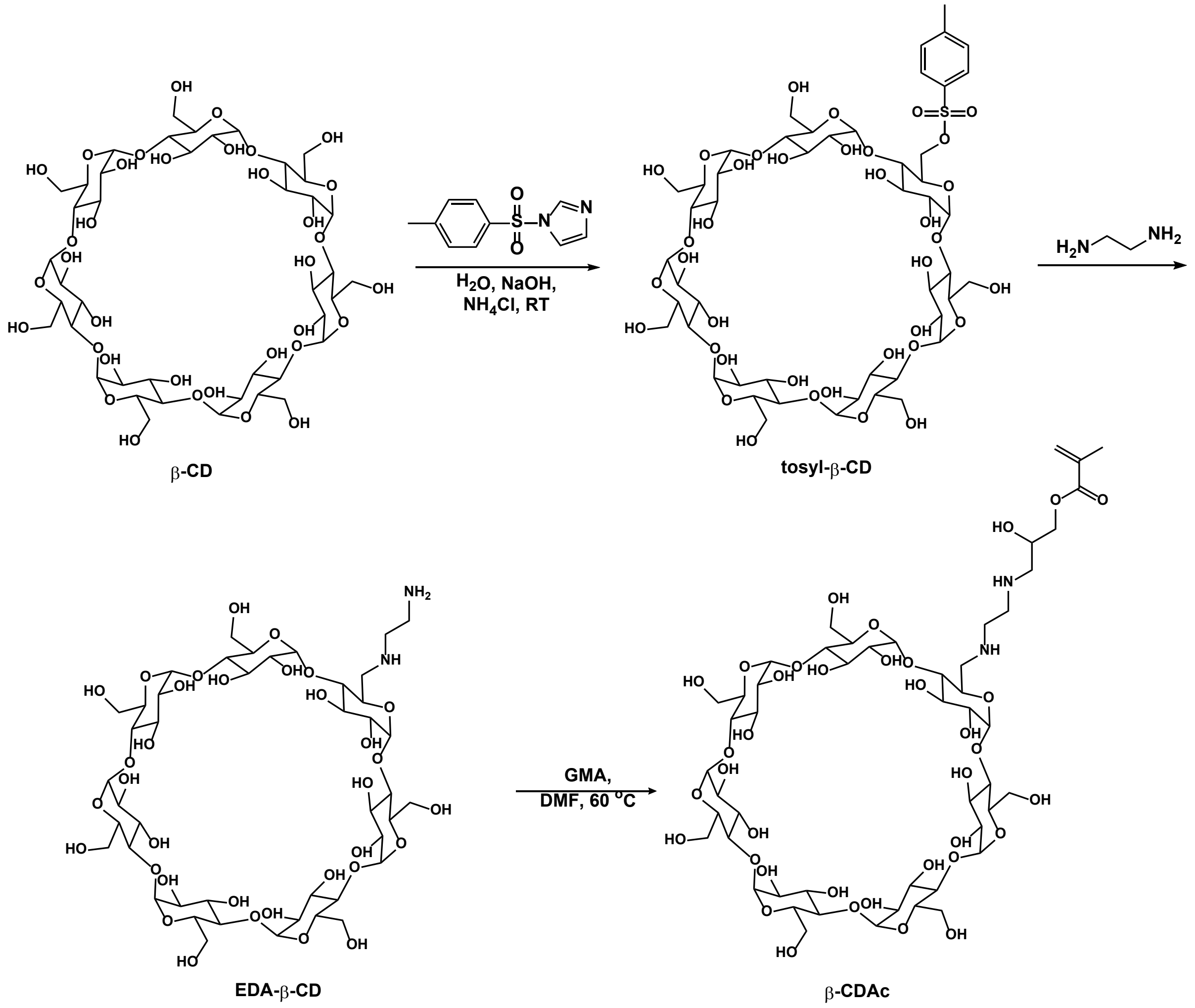

Figure S15. Synthesis route of β-CDAc monomer.

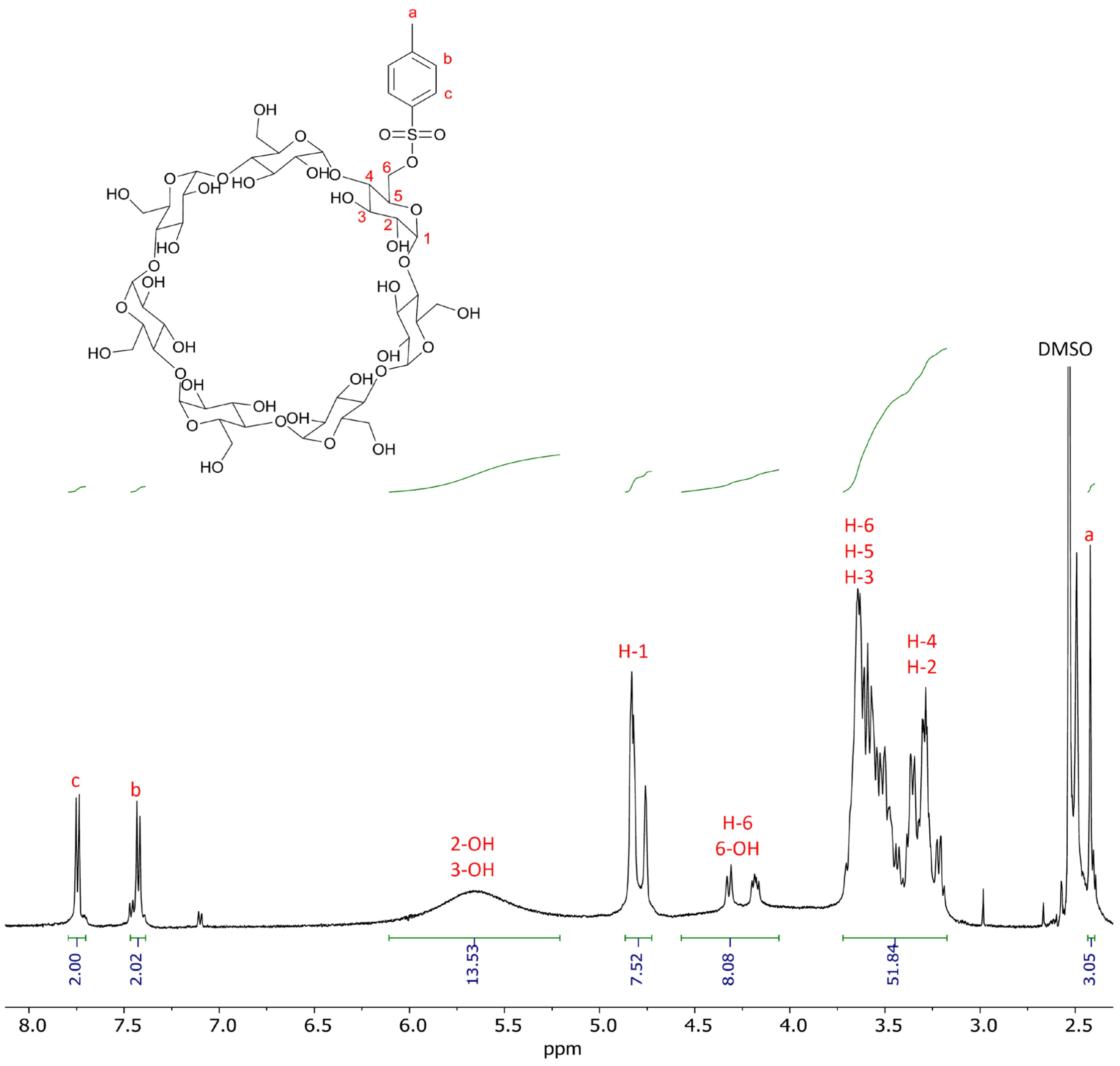

Figure S16. ^{1}H-NMR spectrum of tosyl-β-CD in DMSO-d$_6$.

^{1}H-NMR (500 MHz, DMSO-d$_6$): δ (ppm) 2.41 (s, 3H), 3.17-3.72 (overlap with HDO, m, 40H), 4.05-4.56 (m, 8H), 4.72-4.85 (m, 7H), 5.21-6.10 (br, s, 14H), 7.42 (d, 2 H), 7.74 (d, 2 H)

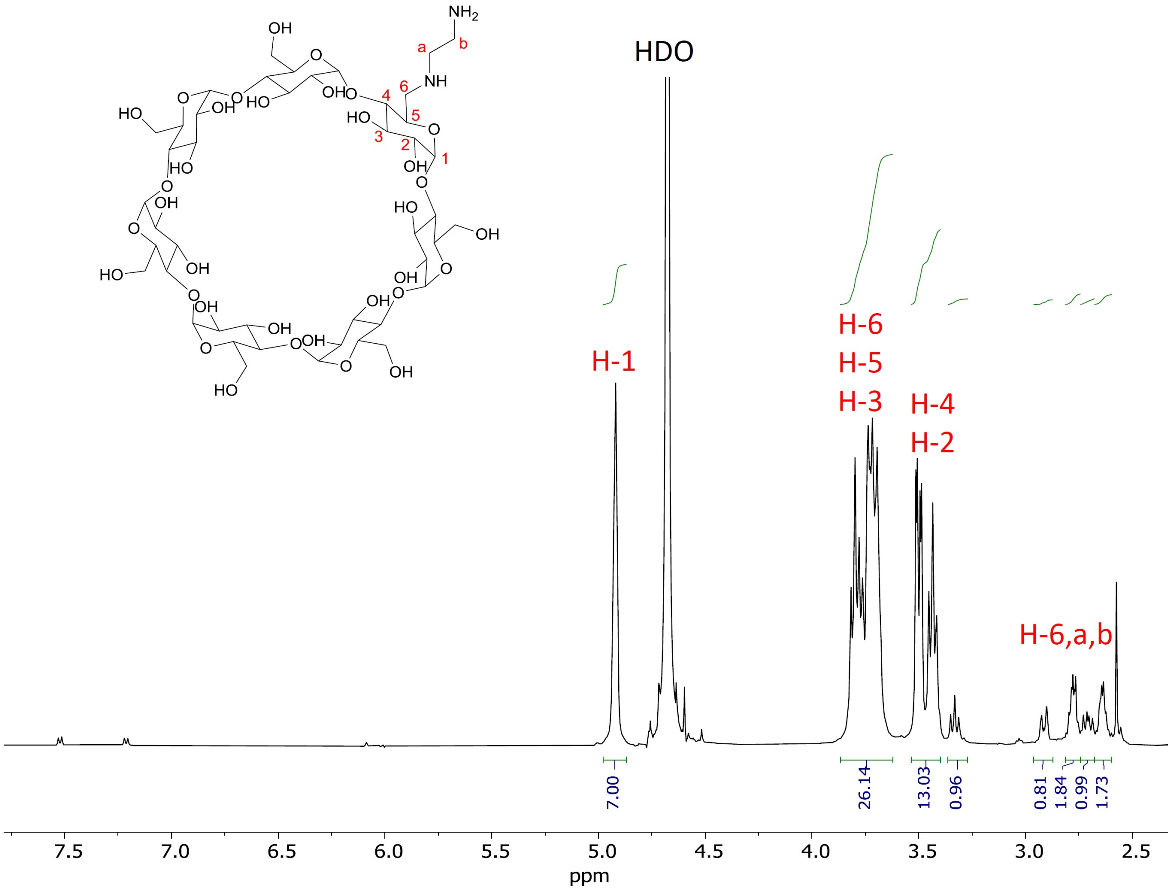

Figure S17. ^{1}H-NMR spectrum of EDA-β-CD in D_2O.

^{1}H-NMR (500 MHz, D_2O): δ (ppm) 2.37-2.95 (m, 6H), 3.33 (t, 1H), 3.39-3.52 (m, 13H), 3.62-3.86 (m, 26H), 4.91 (m, 7H).

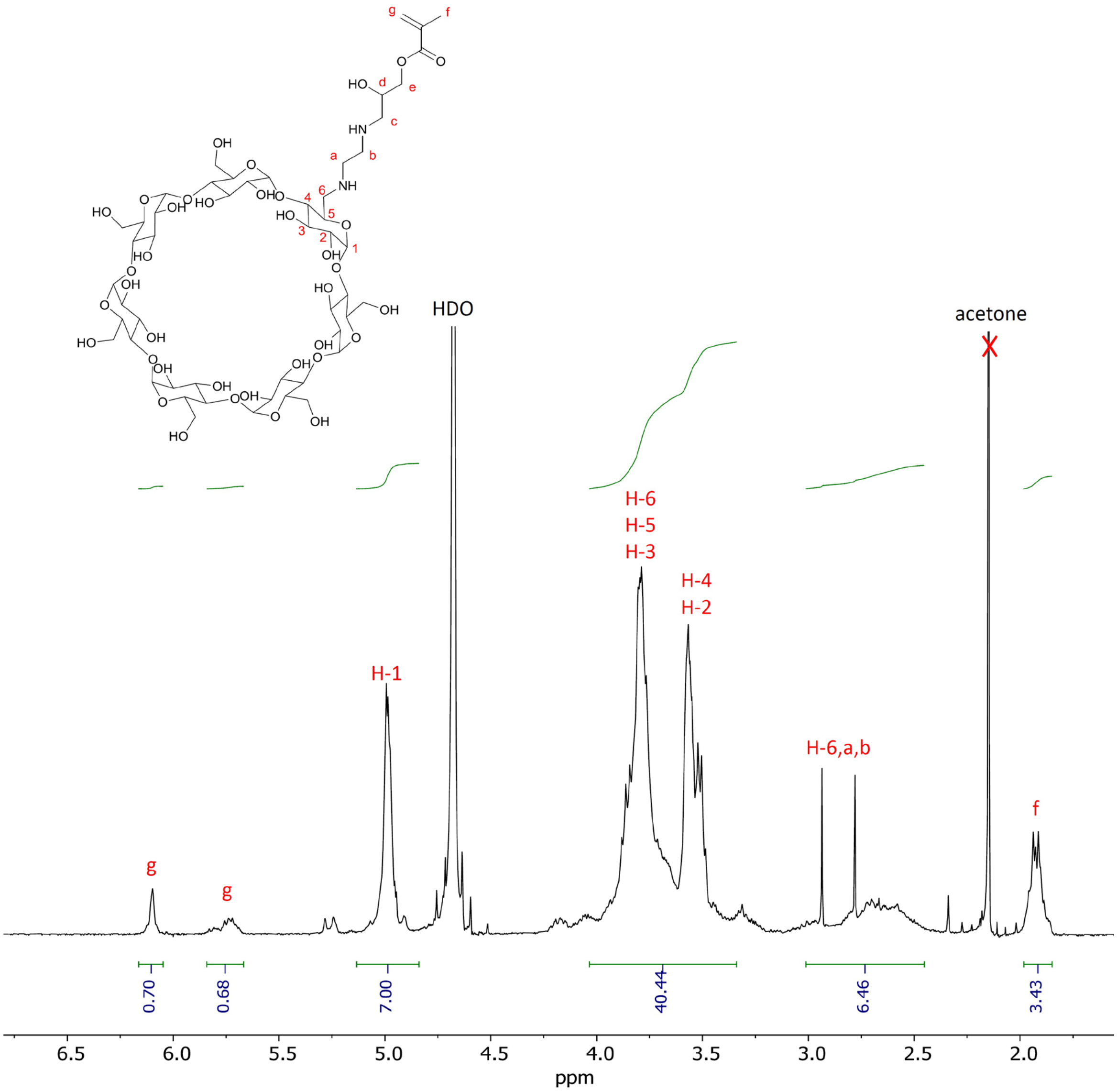

Figure S18. ^{1}H-NMR spectrum of β-CDAc monomer in D_2O.

^{1}H-NMR (500 MHz, D_2O): δ (ppm) 1.92 (m, 3H), 2.44-3.00 (m, 6H), 3.34-4.02 (m, 40H), 4.98 (s, 7H), 5.75 (m, 1H), 6.10 (s, 1H).

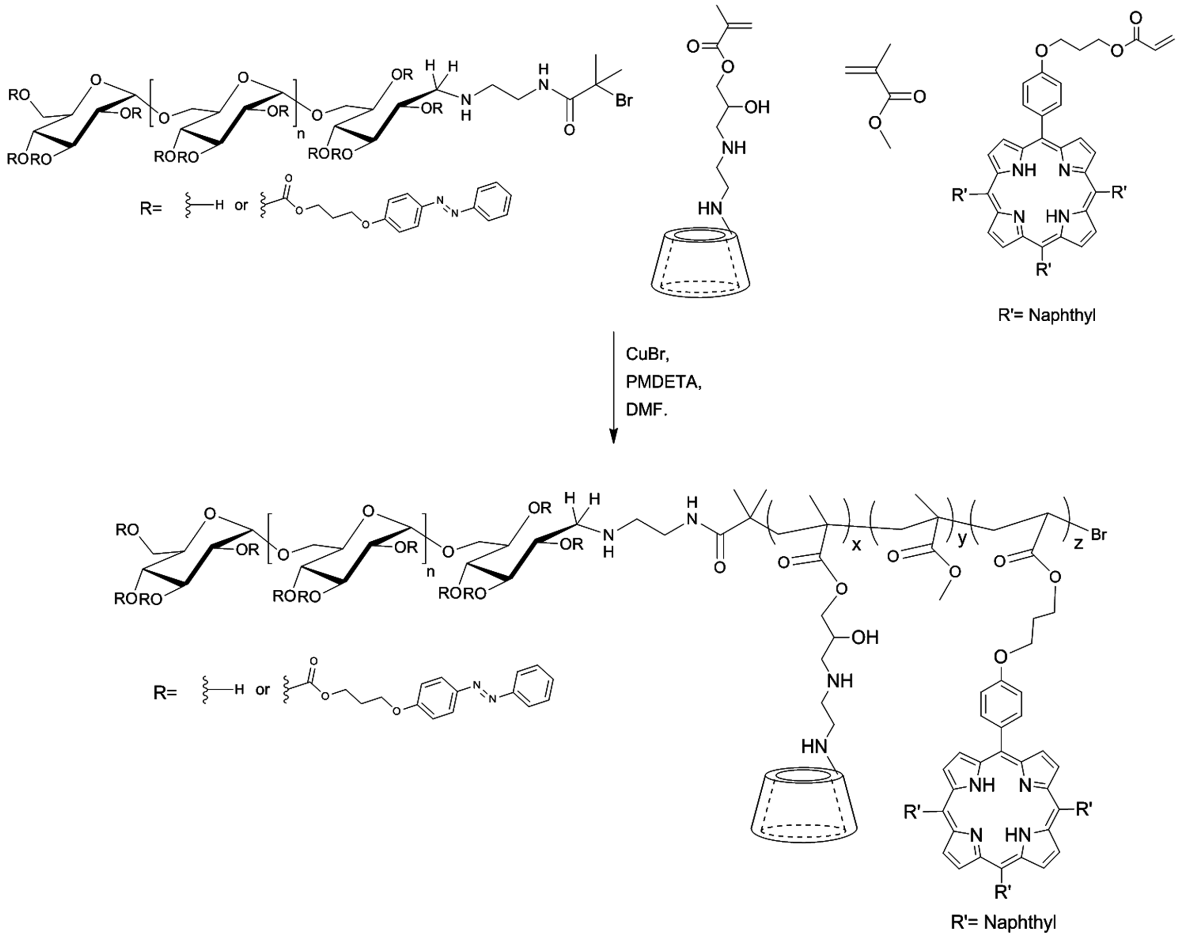

Figure S19. Synthesis of b-(Azo-grafted Dex)-b((MMA)-r-(β-CDAc)-r-(PorAc))

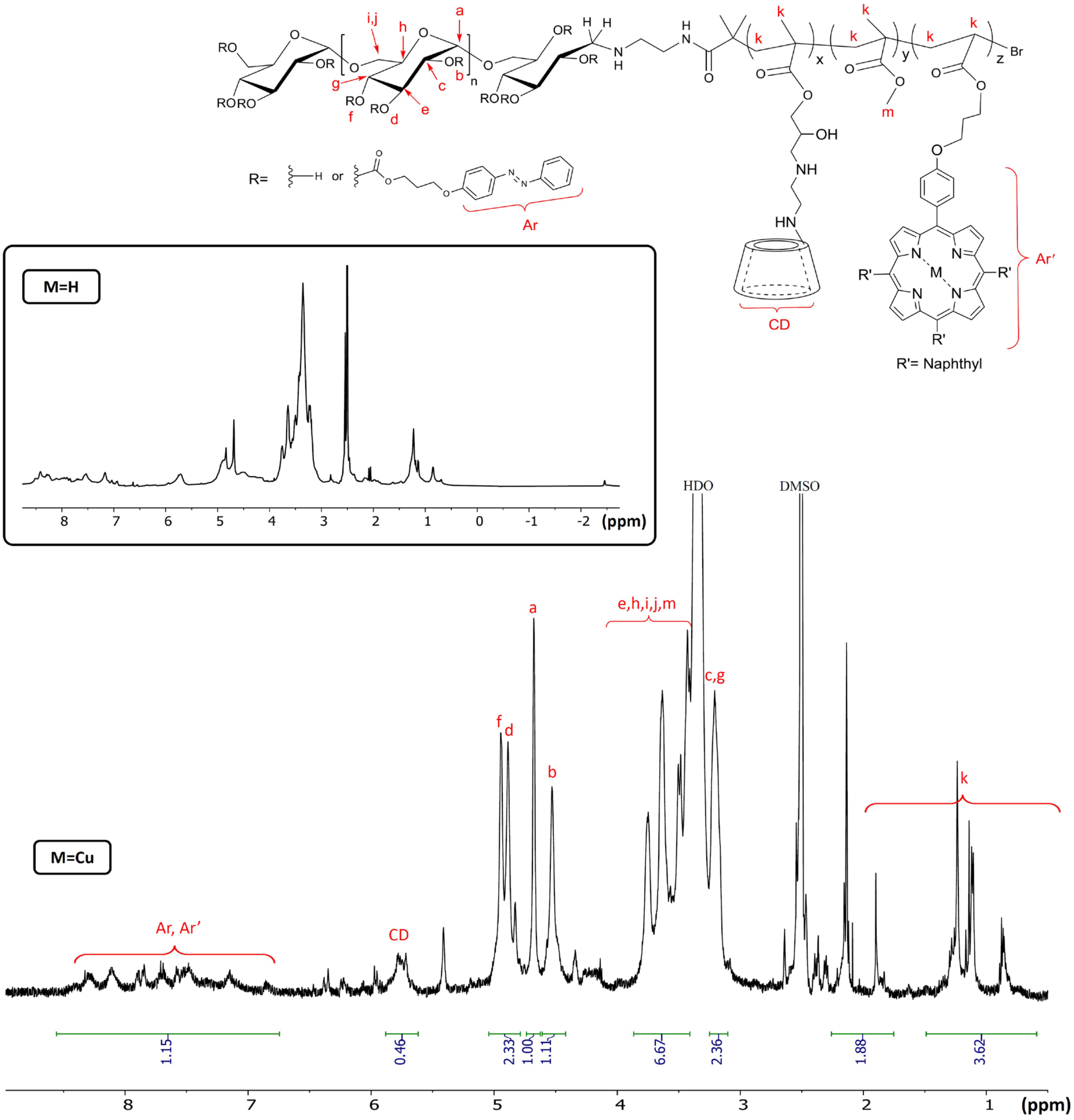

Figure S20. ^{1}H-NMR of b-(Azo-grafted Dex)-b((MMA)-r-(β-CDAc)-r-(PorAc)) in DMSO-d_6

^{1}H-NMR (500 MHz, DMSO-d_6): δ (ppm) 0.59-2.25 (k, C$\boldsymbol{H}_3$, C$\boldsymbol{H}_2$), 3.10-3.25 (c, g), 3.40-3.86 (e, h, i, j, m), 4.41-4.60 (b), 4.62-4.73 (a, Anomeric H), 4.78-5.04 (d, f), 5.63-5.88 (CD), 6.75-8.56 (Aromatic H).

Table S1. Molecular weight and polydispersity index of Dex, Azo-grafted Dex-Br, and b-(Azo-grafted Dex)-b((MMA)-r-(β-CDAc)-r-(PorAc)).

Sample	M_n (kDa)	PDI
**Dex**	18.3	1.37
**Azo-grafted Dex**	21.6	1.44
**b-(Azo-grafted Dex)-b((MMA)-r-(β-CDAc)-r-(PorAc))**	64.6	1.60

Table S2. Drug loading and encapsulation efficiency of drugs in nanoparticles

Nanoparticle	Drug(s)	Weight ratio of Drug(s):nanoparticles	Drug Loading (%)	Encapsulation Efficiency (%)
Single-drug	Q	1:10	4.83	53.1
Single-drug	5-FU	1:10	3.14	35.5
Dual-drug	Q	1:10	2.98	32.6
	5-FU	1:10	3.38	37.3

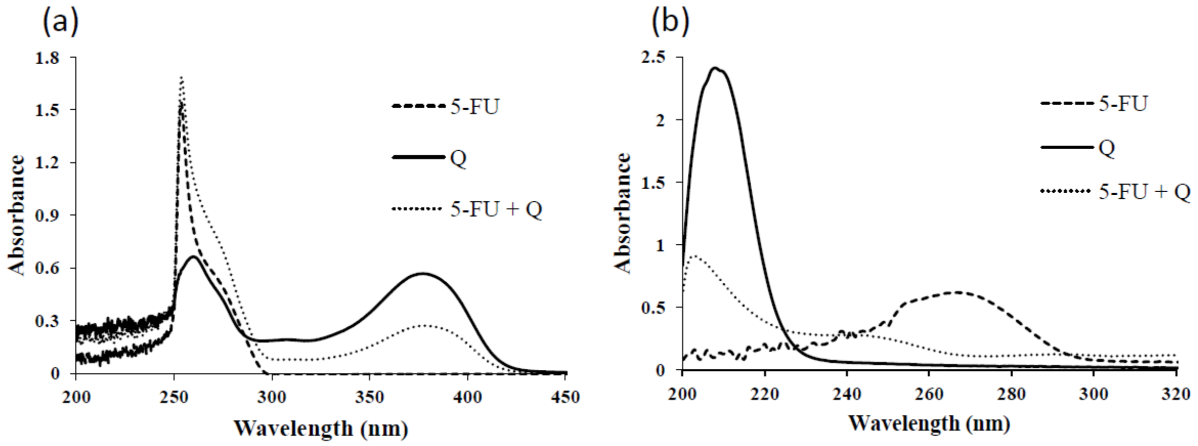

Figure S21. UV-Vis spectra of Quercetin (Q), 5-Fluorouracil (5-FU), and a mix of them in DMSO solvent (a) and in PBS solution (b)
